# Diversity and distribution of blister beetles (Coleoptera, Meloidae) from north-western Saudi Arabia: new observations and first description of a male *Mylabris desertica* Bologna, 2007

**DOI:** 10.3897/BDJ.13.e174504

**Published:** 2025-12-22

**Authors:** Vidak Lakušić, Ladislav Černý, David Kopr, Martina Panisi, Yuri Simone, Marko Stanisavljević, László Patkó, Ayman Abdulkarem, Benjamin P. Y-H. Lee, Magdy El-Bana, Ahmed Al-Ansari, Omar Al-Attas, José Carlos Brito

**Affiliations:** 1 University of Belgrade, Faculty of Biology, Studentski trg 16, 11000, Belgrade, Serbia University of Belgrade, Faculty of Biology, Studentski trg 16, 11000 Belgrade Serbia; 2 CIBIO, Centro de Investigação em Biodiversidade e Recursos Genéticos, InBIO Laboratório Associado, Campus de Vairão, Universidade do Porto, Vairão, Portugal CIBIO, Centro de Investigação em Biodiversidade e Recursos Genéticos, InBIO Laboratório Associado, Campus de Vairão, Universidade do Porto Vairão Portugal; 3 BIOPOLIS Program in Genomics, Biodiversity and Land Planning, CIBIO, Campus de Vairão, Vairão, Portugal BIOPOLIS Program in Genomics, Biodiversity and Land Planning, CIBIO, Campus de Vairão Vairão Portugal; 4 The South Bohemian Museum in České Budějovice, Dukelská 1, CZ-370 51, České Budějovice, Czech Republic The South Bohemian Museum in České Budějovice, Dukelská 1, CZ-370 51 České Budějovice Czech Republic; 5 Faculty of Agrisciences, Department of Zoology, Mendel University, Zemědělská 1, Brno, Czech Republic Faculty of Agrisciences, Department of Zoology, Mendel University, Zemědělská 1 Brno Czech Republic; 6 University of Antwerpen, Laboratory of Functional Morphology, Antwerp, Belgium University of Antwerpen, Laboratory of Functional Morphology Antwerp Belgium; 7 The Royal Commission for AlUla, Oud Dunes - Amr Aldamri, Riyadh 07747, Saudi Arabia The Royal Commission for AlUla, Oud Dunes - Amr Aldamri Riyadh 07747 Saudi Arabia; 8 Department of Environmental Protection and Regeneration, Red Sea Global (RSG - Red Sea Zone), Umluj, Saudi Arabia Department of Environmental Protection and Regeneration, Red Sea Global (RSG - Red Sea Zone) Umluj Saudi Arabia; 9 Faculty of Science, Port Said University, Port Said, Egypt Faculty of Science, Port Said University Port Said Egypt; 10 Departmant of Environment, Faculty of Environmental Sciences, King Abdulaziz University, Jeddah, Saudi Arabia Departmant of Environment, Faculty of Environmental Sciences, King Abdulaziz University Jeddah Saudi Arabia; 11 Departamento de Biologia, Faculdade de Ciências, Universidade do Porto, Porto, Portugal Departamento de Biologia, Faculdade de Ciências, Universidade do Porto Porto Portugal

**Keywords:** Arabian Peninsula, Middle East, desert fauna, new records, arid environment

## Abstract

**Background:**

This study presents the first faunistic records of blister beetles (Coleoptera, Meloidae) from north-western Saudi Arabia, specifically Hejaz mountains and north Tihama, expanding the known distribution range of the species within the country. Field surveys were conducted between 2022 and 2024, aiming to cover different seasons. These observations contribute to the knowledge of the diversity of meloid fauna of the country and underscoring the importance of continued faunistic exploration in north-western Saudi Arabia, a region that remains relatively under-surveyed in terms of meloid diversity.

**New information:**

Three species (*Hycleus borchmannianus*, *Hycleus trizonatus*, *Mylabris filicornis*) and one genus (*Sitaris*) are recorded for the first time in Saudi Arabia. Besides that, this work also provides the first description of the male morphology and genitalia of *Mylabris desertica* Bologna, 2007.

## Introduction

The north-western region of Saudi Arabia remains one of the least explored areas of the country in terms of insect biodiversity, with many insect groups still lacking comprehensive taxonomic studies or recent revisions. Notably, no comprehensive surveys or studies have focused on beetles (Coleoptera) from this part of the Kingdom. The present study represents the first systematic investigation of beetles from the family Meloidae (blister beetles), in north-western Saudi Arabia and, to our knowledge, the first systematic study of any beetle group from this region.

The first comprehensive work on the family Meloidae from the Arabian Peninsula was written by Kaszab (1983), who also described one genus, 16 species and three subspecies that were new to science. The list of species occurring in Saudi Arabia was subsequently expanded by Schneider (1991), who described one new species and provided several new distribution records. The most recent and extensive checklist for the Arabian Peninsula was provided by Bologna and Turco (2007), who reviewed all previously published literature and described five new species. Since then, one new species of *Mesomeloe* has been described from Qatar (Sánchez‐Vialas et al. 2021a). According to their works, a total of 88 species and 10 subspecies of Meloidae have been recorded from the Arabian Peninsula, of which 75 species and nine subspecies occur in Saudi Arabia. Since the publication of their checklists, only eight additional records from Saudi Arabia have been published (El-Hawagry et al. 2013, El-Hawagry et al. 2015, Abdel-Dayem et al. 2017, Abdel-Dayem et al. 2020, Alzahrani et al. 2025). The majority of the existing knowledge on the Meloidae of the Arabian Peninsula is restricted to its south-western region, while the Hejaz mountain range and northern Tihama still remain largely unexplored. This geographic gap highlights the potential for the discovery of additional species and the expansion of the current checklist of Arabian meloids (Bologna and Turco 2007).

Within the family Meloidae, Mylabris (Mylabris) desertica was described by Bologna and Turco (2007) from a single, partly damaged, female specimen collected from Saudi Arabia, thus leaving the male morphological features undescribed. When only the female of a species is known, describing the male becomes essential for achieving a complete taxonomic characterisation. In many insect groups, including Meloidae, male genitalia represent the most stable and diagnostically informative morphological trait, often exhibiting clear interspecific differences that enable confident identification (Song and Bucheli 2010). Documenting the male morphology not only resolves existing taxonomic gaps, but also provides a foundation for further taxonomic revisions and phylogenetic studies within the group.

This work helps to fill the significant biogeographic and taxonomic gaps in north-western Saudi Arabia and provides critical insights into the diversity, distribution and conservation needs of the local insect fauna. New distributional records of species from Meloidae family are presented, including first records of three species and one genus in the country. Finally, the study provides the first description of the male of *M. desertica*, providing photographs and drawings of diagnostic characters.

## Materials and methods

**Study area**

The study area, encompassing approximately 97,605 km², is situated in the north-western region of the Saudi Arabia. Topographically, elevation varies markedly, ranging from -6 metres along the Red Sea coastline to a maximum of 1,958 metres at Khaybar White Volcano in the south-eastern sector. The landscape is predominantly shaped by the Hijaz Mountain range, which comprises several prominent volcanic and mountainous formations exceeding 1,000 metres in elevation. Wetlands are mostly absent from the study area and, in the mountain regions, they are scarce, predominantly isolated and ephemeral. Wadi Al Hamd in the southwest is the only permanent water source (Lehner et al. 2008).

Although the study region is predominantly arid, there is a pronounced longitudinal gradient in annual precipitation (Fick and Hijmans 2017). Western coastal zones exhibit extremely low precipitation levels, whereas precipitation increases markedly towards the interior. Temperature patterns tend to follow the altitudinal variation, with higher mean annual temperatures (26.8°C) recorded in southern lowland coastal areas and lower temperatures (16.2°C) in northern and highland inland regions. The majority of the region is classified as hyper-arid, with only the mountain peaks and eastern zones classified as within the arid category (Trabucco and Zomer 2018).

**Figure 1.**
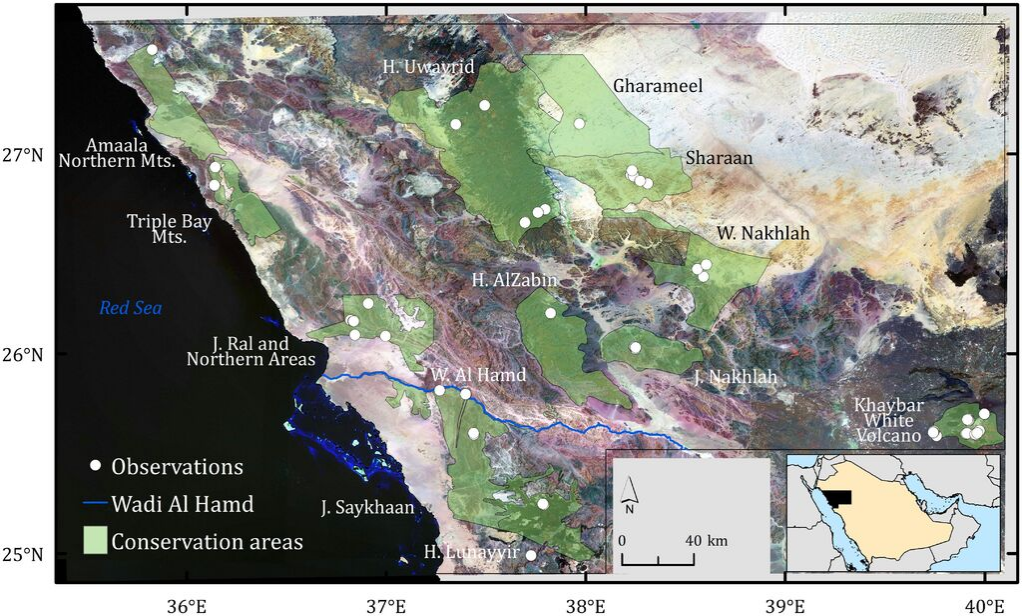
Localities of records of Meloidae (n = 75) in the study area and location of the study area in the north-western Saudi Arabia (small inset).

Fieldwork was conducted in 13 conservation areas managed by The Royal Commission for AlUla and the Red Sea Global, including one Biosphere Reserve (Harrat Uwayrid), one Geopark (Khaybar White Volcano), one National Park (Sharaan), three Nature Reserves (Gharameel, Harrat AlZabin, Wadi Nakhlah), one Area Rich in Biodiversity (Jabal Nakhlah) and six local Key Biodiversity Areas (Amaala Northern Mountains, Jabal Ral and Northern Areas, Jabal Saykhaan, Harrat Lunayyir, Triple Bay Mountains and Wadi Al Hamd) (UNEP-WCMC 2019, El-Bana et al. 2025) (Fig. 1).

**Data sampling and analysis**

Fieldwork was conducted between November 2022 and October 2024. During this period, seven field missions were carried out, lasting for 230 days in total, covering various seasonal and environmental conditions spread across 231 different sites. Collecting was performed by random walks, covering different types of microhabitats in each site. Most of the species were collected on flowering plants during the daytime, some were instead found on the ground during the night (*Mesomeloe coelatus* and *Eurymeloe saharensis)* and some were attracted by the headlamp or light trap (*Lydomorphus* spp. *a*nd Zonitoschema spp.). Specimens were collected by hand and preserved in ethanol (99%) for morphological investigation. Geographic coordinates were taken with a GPS using the WGS84 datum. Photos of specimens in the field were taken with an Olympus tough TG-6.

Collected specimens were mounted on mounting boards or directly pinned through the right elytron. Photographs of whole specimens were taken using a Nikon D5300 digital camera, equipped with a Tamron SP Di AF 90 mm F/2.8 macro lens and a Sigma EM-140 DG ring flash with a DIY light diffuser. Digital images of *Mylabris desertica* were taken using a Canon EOS 750D with the Canon MP-E 65 mm macrolens. The specimens are deposited in the collection of AlUla Museum and Jihočeské muzeum v Českých Budějovicích [The South Bohemian Museum in České Budějovice] (JMBC) with URN numbers not yet assigned.

Identification of specimens was performed by the second author. The taxonomic arrangement is used according to Bologna (2020), except for Meloini where Sánchez‐Vialas et al. (2021b) and Sánchez-Vialas et al. (2022) were followed. Sánchez‐Vialas et al. (2021b) were relied upon because they presented a fully supported molecular phylogeny and clear evidence that genus *Meloe* Linnaeus, 1758 is paraphyletic and should be divided into several genera. However, Riccieri et al. (2024) did not accept this elevation, with the argument based on larval and adult morphology and preliminary molecular evidence which are part of ongoing, unpublished work, meaning that no currently available data justify departing from the conclusions of Sánchez‐Vialas et al. (2021b). Species *Zonitoschema iranica* was identified, based on Batelka and Bologna (2014). The complete dataset is available in Suppl. material 1.

## Taxon treatments

### Mylabris (Mylabris) desertica

Bologna, 2007

8CA4285C-A45A-5AC5-B1C6-113472447A87

#### Materials

- **Type status:** Other material. **Occurrence:** recordedBy: Lakušić Vidak; associatedReferences: VLA_4104; occurrenceID: D867E344-30D9-520C-AF61-0A72FF8F8FAE; **Location:** country: Saudi Arabia; stateProvince: Tabuk; municipality: Umluj; locality: 45km E of Umluj; maximumElevationInMeters: 721; verbatimCoordinates: 24.990833N, 37.725556E; **Event:** eventDate: 18.03.2024.

#### Description

**Figure 2.**
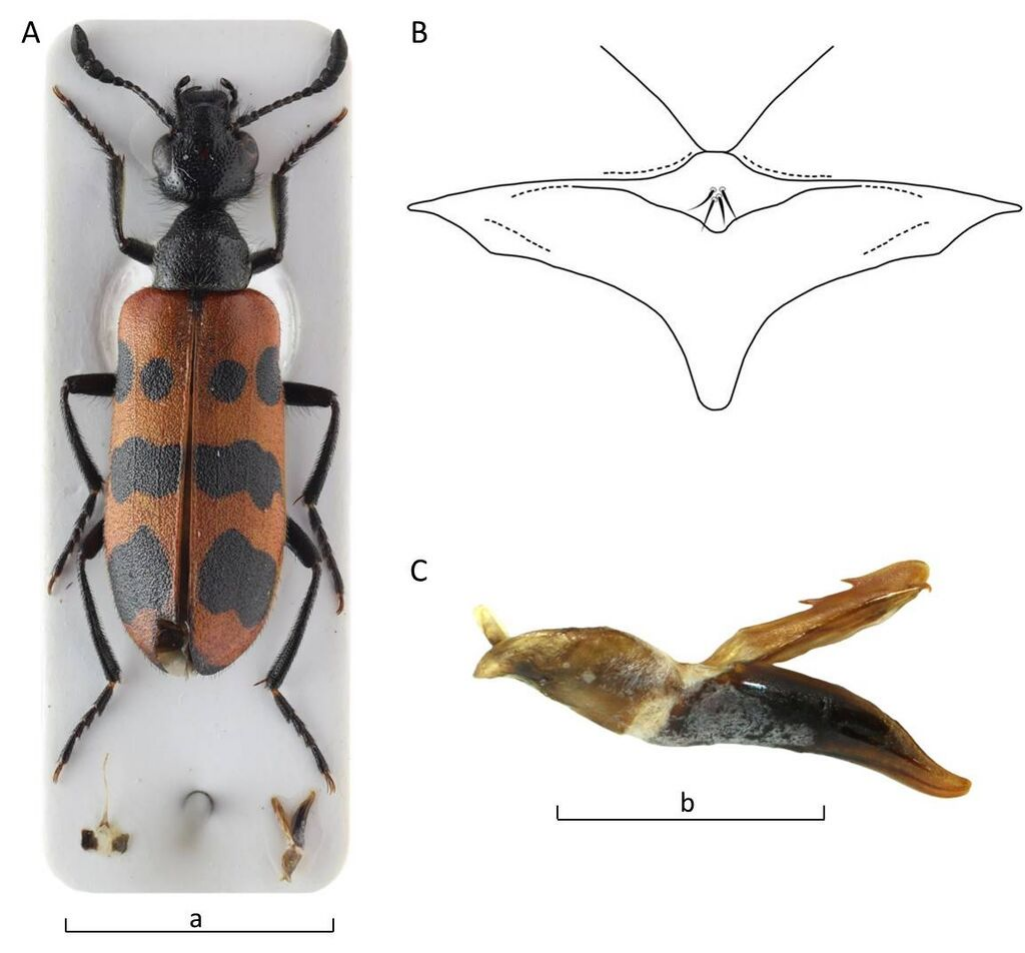
*Mylabris desertica* (VLA_4104): **A** habitus; **B** mesosternal sclerite; **C** aedeagus. Bar size a = 5 mm, b = 1 mm. Photo credits Ladislav Černý.

Length: 11.5 mm (Fig. 2). Body uniformly black, the macrosetation is mixed black and white, with white predominating on the abdomen and ventral side of the thoracic segments.

*Head* is black, frons with a red spot. Head capsule as long as wide with large and sparse punctures, except for the frontal area, where it is dense; surrounding surface smooth and shiny. Temples parallel and rounded posteriorly, shorter than eyes in lateral view. Macrosetation of the head long and erect, black. Clypeus transverse, wider than long, convex with fore margin rounded. Apical third is smooth, the rest rugose, strongly punctured to wrinkled, fronto-clypeal suture clearly visible. Labrum as long as clypeus, wider, anterior margin slightly emarginate, longitudinally depressed in the middle, almost smooth with long black setation, except fore margin, where it is short and whitish. Mandibles well developed with long macrosetation laterally, not regularly convex, but bent at the middle and at apex, longer than clypeus and labrum together. Maxillary palpomeres with long setae, last maxillary palpomeres are obtusely pointed. The eyes are broadly oval. Antennae black, composed of eleven, progressivelly widened, well-separated segments. The last four segments (VIII-XI) clearly wider than the others. Antennomeres I–II with long macrosetation, black; III–VII with short macrosetation, shiny with faint reddish-brown tinge; antennomere III 1.4x as long as IV; antennomere XI 1.5x as long as X and tapering to the faint tip.

*Pronotum* is longer than wide, tapered in front, with a distinct dorsal depression; macrosetation long, erect, black mixed whitish; with large punctures, laterally and posteriorly contiguous. Mesosternum with developed scutum with a few distinctive macrosetae in its posterior part (Fig. 2B).

*Elytra* covered with sparse, black and white and slightly inclined macrosetae. The black pattern of each elytron consists of short black emargination of the suture behind the scutellum; of two sub-basal black spots, sutural and humeral, the humeral is larger; a transverse medial black spot closely spaced to the suture, but not reaching the outer margin; an irregular postmedian black spot not reaching the margins; and the apex is narrowly black (Fig. 2A).

*Legs* are black with black setation, except for the fore tibiae, where it is mixed with short whitish setation inwardly. Longer black setae are dorsally and laterally on protarsomeres I–IV. Tibial spurs pointed, except the metatibial outer one, which is obtusely ended. The blades of tarsal claws are smooth without denticulation or microcrenulation and the ventral ones are well developed, sharply pointed and reaching the length of the dorsal ones.

*Male genitalia*. Aedeagus (Fig. 2C) with two subequal dorsal hooks, positioned in the middle of the distal half, closely spaced and of equal inclination in *isoharpagae* form. Endophallic hook slender and curved apically. Parameres are slender, concave in lateral view and ca. 2x as long as phallobase, with a pointed and slightly curved apex.

## Checklists

### Checklist of Meloidae of north-western Saudi Arabia

#### Alosimus syriacus syriacus

(Linnaeus, 1758)

ECA6EE64-B3D7-5E5F-B503-8EC21BC3B406

##### Materials

- **Type status:** Other material. **Occurrence:** associatedReferences: VLA_0846; occurrenceID: ECD45199-BE0E-50FA-BF6F-4FB83E3D893B; **Location:** country: Saudi Arabia; locality: Harrat Uwayrid; **Event:** eventDate: 19/2/2023
- **Type status:** Other material. **Occurrence:** associatedReferences: VLA_0852; occurrenceID: 8F0A9AE3-3BE8-57CC-A8FA-C0BDD5F76C55; **Location:** country: Saudi Arabia; locality: Harrat Uwayrid; **Event:** eventDate: 19/2/2023
- **Type status:** Other material. **Occurrence:** associatedReferences: VLA_0901; occurrenceID: DD9EF31A-FBB2-5D6C-9D78-0B39D7E4723B; **Location:** country: Saudi Arabia; locality: Harrat Uwayrid; **Event:** eventDate: 21/2/2023
- **Type status:** Other material. **Occurrence:** associatedReferences: VLA_1142; occurrenceID: 2EDDE188-6342-5F4F-ADAC-166BBEB0ED31; **Location:** country: Saudi Arabia; locality: Khaybar White Volcano; **Event:** eventDate: 3/3/2023
- **Type status:** Other material. **Occurrence:** associatedReferences: VLA_1148; occurrenceID: 43E1E9C1-E4E4-59A5-9263-FD43DE0C0536; **Location:** country: Saudi Arabia; locality: Khaybar White Volcano; **Event:** eventDate: 3/3/2023
- **Type status:** Other material. **Occurrence:** associatedReferences: VLA_1210; occurrenceID: 44E87172-C885-552D-A1B5-3860246FFDF9; **Location:** country: Saudi Arabia; locality: Khaybar White Volcano; **Event:** eventDate: 4/3/2023

##### Diagnosis

Suppl. material 2.

#### Lydomorphus angusticollis suturellus

(Haag-Rutenberg, 1880)

925E871B-481E-5541-8DC3-C78C1E8A9465

##### Materials

- **Type status:** Other material. **Occurrence:** associatedReferences: VLA_3325; occurrenceID: 753FD154-6CE0-55C7-8608-C99649BF8CB3; **Location:** country: Saudi Arabia; locality: Jabal Ral and Northern Areas; **Event:** eventDate: 5/4/2024
- **Type status:** Other material. **Occurrence:** associatedReferences: VLA_3371; occurrenceID: 6B194808-BB16-5194-874A-18E5ECF1B07B; **Location:** country: Saudi Arabia; locality: Jabal Ral and Northern Areas; **Event:** eventDate: 7/4/2024

##### Diagnosis

Suppl. material 3.

#### Lydomorphus brittoni

(Kaszab, 1953)

D557BADD-081C-5FC4-B858-27FE33874252

##### Materials

- **Type status:** Other material. **Occurrence:** associatedReferences: VLA_1232; occurrenceID: 5C73E8ED-33C1-5C86-80A9-9564B3FB1E23; **Location:** country: Saudi Arabia; locality: Khaybar White Volcano; **Event:** eventDate: 4/3/2023
- **Type status:** Other material. **Occurrence:** associatedReferences: VLA_1495; occurrenceID: 48AA5C65-F309-5126-B2AD-7A7146C25781; **Location:** country: Saudi Arabia; locality: Khaybar White Volcano; **Event:** eventDate: 8/5/2023
- **Type status:** Other material. **Occurrence:** associatedReferences: VLA_1614; occurrenceID: 0274FE1B-6CBB-563F-8D91-B5C6B1480E41; **Location:** country: Saudi Arabia; locality: Khaybar White Volcano; **Event:** eventDate: 11/5/2023

##### Diagnosis

Suppl. material 4.

#### Lydomorphus palaestinus

(Kirsch, 1870)

00F89E8C-F242-525F-BD81-EFFF55F68E66

##### Materials

- **Type status:** Other material. **Occurrence:** associatedReferences: VLA_0957; occurrenceID: 185D18D3-6898-5DF8-B954-455CBF3BD913; **Location:** country: Saudi Arabia; locality: Sharaan; **Event:** eventDate: 24/2/2023
- **Type status:** Other material. **Occurrence:** associatedReferences: VLA_0994; occurrenceID: C4BFB526-C6C4-5661-958C-C983EAC30021; **Location:** country: Saudi Arabia; locality: Sharaan; **Event:** eventDate: 25/2/2023

##### Diagnosis

Suppl. material 5.

#### Ammabris elegans

Olivier, 1811

E3E23220-6789-5741-BF2A-DA829E0CE35A

##### Materials

- **Type status:** Other material. **Occurrence:** associatedReferences: VLA_1812; occurrenceID: 039CDC51-0C69-5D08-AD36-AC61CE7582BA; **Location:** country: Saudi Arabia; locality: Sharaan; **Event:** eventDate: 24/5/2023

##### Diagnosis

Suppl. material 6.

#### Croscherichia goryi

(Marseul, 1870)

2FE95911-BE02-5654-811C-4466041EB70F

##### Materials

- **Type status:** Other material. **Occurrence:** associatedReferences: VLA_1868; occurrenceID: DC12F81D-832C-51B8-9262-04806D0FC002; **Location:** country: Saudi Arabia; locality: Sharaan; **Event:** eventDate: 26/5/2023
- **Type status:** Other material. **Occurrence:** associatedReferences: VLA_1872; occurrenceID: 01B48321-8166-545A-B5E2-7FA5E2E4A95D; **Location:** country: Saudi Arabia; locality: Sharaan; **Event:** eventDate: 26/5/2023
- **Type status:** Other material. **Occurrence:** associatedReferences: VLA_1873; occurrenceID: 4564FD9D-D095-5EE9-8DF5-1E36ECE86D5B; **Location:** country: Saudi Arabia; locality: Sharaan; **Event:** eventDate: 26/5/2023
- **Type status:** Other material. **Occurrence:** associatedReferences: VLA_3599; occurrenceID: 934C4BD1-E964-5A78-B394-82EB97807139; **Location:** country: Saudi Arabia; locality: Harrat Lunayyir; **Event:** eventDate: 21/4/2024

##### Diagnosis

Suppl. material 7.

#### Croscherichia sanguinolenta arabica

Bologna, 1991

4F0E2023-CA1A-5995-A665-FA8484BABD10

##### Materials

- **Type status:** Other material. **Occurrence:** associatedReferences: VLA_1541; occurrenceID: A073816E-823A-5E33-A12A-24C13C1C7CCC; **Location:** country: Saudi Arabia; locality: Khaybar White Volcano; **Event:** eventDate: 10/5/2023
- **Type status:** Other material. **Occurrence:** associatedReferences: VLA_1542; occurrenceID: FC767F31-8592-518B-994C-F486CC396081; **Location:** country: Saudi Arabia; locality: Khaybar White Volcano; **Event:** eventDate: 10/5/2023

##### Diagnosis

Suppl. material 8.

#### Croscherichia tigrinipennis

(Latreille, 1827)

8B48BBAA-DC76-5ED4-B161-C6ED6F434ACF

##### Materials

- **Type status:** Other material. **Occurrence:** associatedReferences: VLA_3125; occurrenceID: F92AF415-B622-578D-B430-E5DDC6903915; **Location:** country: Saudi Arabia; locality: Jabal Saykhaan; **Event:** eventDate: 15/3/2024

##### Diagnosis

Suppl. material 9.

#### Hycleus borchmannianus

(Kaszab, 1983)

4497E232-09AC-548D-8577-FF539FEA4113

##### Materials

- **Type status:** Other material. **Occurrence:** associatedReferences: VLA_1627; occurrenceID: AE5B9480-DF34-5DE9-A1D9-07A916C87F02; **Location:** country: Saudi Arabia; locality: Khaybar White Volcano; **Event:** eventDate: 12/5/2023

##### Notes

**Figure 3.**
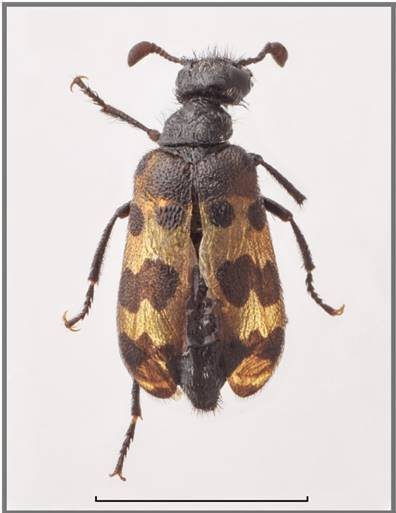
*Hycleus borhcmanianus* (VLA_1627). Bar size = 5 mm. Photo credits Nikola Vesović.

This is the first record for Saudi Arabia, previously considered endemic to Yemen (Fig. 3).

##### Diagnosis

Suppl. material 10.

#### Hycleus novemdecimpunctatus

(Olivier, 1811)

E6DE223C-0BEE-5AFD-A150-55EE4A75B7EC

##### Materials

- **Type status:** Other material. **Occurrence:** associatedReferences: VLA_1506; occurrenceID: CCFF0C76-B2C0-5A21-A36F-CA95B0199D8B; **Location:** country: Saudi Arabia; locality: Khaybar White Volcano; **Event:** eventDate: 9/5/2023
- **Type status:** Other material. **Occurrence:** associatedReferences: VLA_1547; occurrenceID: 878FD6CD-6158-53FE-A1D4-60D6127BAE39; **Location:** country: Saudi Arabia; locality: Khaybar White Volcano; **Event:** eventDate: 10/5/2023
- **Type status:** Other material. **Occurrence:** associatedReferences: VLA_1608; occurrenceID: 6DC0FA0E-4F81-56E6-BDBB-3BDFEE405E42; **Location:** country: Saudi Arabia; locality: Khaybar White Volcano; **Event:** eventDate: 11/5/2023
- **Type status:** Other material. **Occurrence:** associatedReferences: VLA_1624; occurrenceID: 6D8E2759-985F-5E5F-A201-D3B336C69CE5; **Location:** country: Saudi Arabia; locality: Khaybar White Volcano; **Event:** eventDate: 12/5/2023

##### Notes

Taxonomic decision published by Pardo-Alcaide (1954) was adopted, which is in contrast to Kaszab (1983) who considered this species to be *H. semifasciatus* (Pic, 1895), currently listed as a junior subjective synonym of *H. novemdecimpunctatus* (*Bologna 2020*). In fact, *H. semifasciatus* differs from the original description of *H. novemdecimpunctatus* (Olivier 1811) due to the absence of a black scutellar spot, which also does not occur in the material collected nor in all the material analysed from other areas of its distribution (Morocco, Tunisia, Israel to Saudi Arabia).

##### Diagnosis

Suppl. material 11.

#### Hycleus pseudobrunnipes

(Kaszab, 1983)

14173B9F-290C-51FB-857C-177DC5771535

##### Materials

- **Type status:** Other material. **Occurrence:** associatedReferences: VLA_0523; occurrenceID: 0F4B870B-6884-5262-999D-A27B5E8952C9; **Location:** country: Saudi Arabia; locality: Jabal Nakhlah; **Event:** eventDate: 2/2/2023
- **Type status:** Other material. **Occurrence:** associatedReferences: VLA_1512; occurrenceID: BA6AC022-0275-54B9-8A12-2294F97987FD; **Location:** country: Saudi Arabia; locality: Khaybar White Volcano; **Event:** eventDate: 9/5/2023
- **Type status:** Other material. **Occurrence:** associatedReferences: VLA_3282; occurrenceID: D385B396-468E-501D-A2F3-BF41B70187B3; **Location:** country: Saudi Arabia; locality: Jabal Ral and Northern Areas; **Event:** eventDate: 4/4/2024
- **Type status:** Other material. **Occurrence:** associatedReferences: VLA_3283; occurrenceID: 327EDAE2-43A1-5618-B79B-FC0E598163D3; **Location:** country: Saudi Arabia; locality: Jabal Ral and Northern Areas; **Event:** eventDate: 4/4/2024
- **Type status:** Other material. **Occurrence:** associatedReferences: VLA_3319; occurrenceID: D271D2B0-B973-5B2B-9803-08E984DB0A75; **Location:** country: Saudi Arabia; locality: Jabal Ral and Northern Areas; **Event:** eventDate: 5/4/2024
- **Type status:** Other material. **Occurrence:** associatedReferences: VLA_3330; occurrenceID: F3B2A034-7133-5ABE-9C66-5D408C9D407D; **Location:** country: Saudi Arabia; locality: Jabal Ral and Northern Areas; **Event:** eventDate: 6/4/2024
- **Type status:** Other material. **Occurrence:** associatedReferences: VLA_3604; occurrenceID: 0905F7B5-FA7D-5F61-A6F7-41D57EF8B539; **Location:** country: Saudi Arabia; locality: Harrat Lunayyir; **Event:** eventDate: 22/4/2024

##### Diagnosis

This species exhibits considerable morphological variability, particularly in the elytral pattern and body size. The elytral markings can consist of isolated spots, which often merge to form partially transverse irregular bands (Suppl. material 12).

#### Hycleus scabratus

(Klug, 1834)

077752F3-7525-5B35-A935-EF2C53CC69EC

##### Materials

- **Type status:** Other material. **Occurrence:** associatedReferences: VLA_1548; occurrenceID: B887F732-5325-55D2-B7F1-555041ED9970; **Location:** country: Saudi Arabia; locality: Khaybar White Volcano; **Event:** eventDate: 10/5/2023

##### Diagnosis

**Figure 4.**
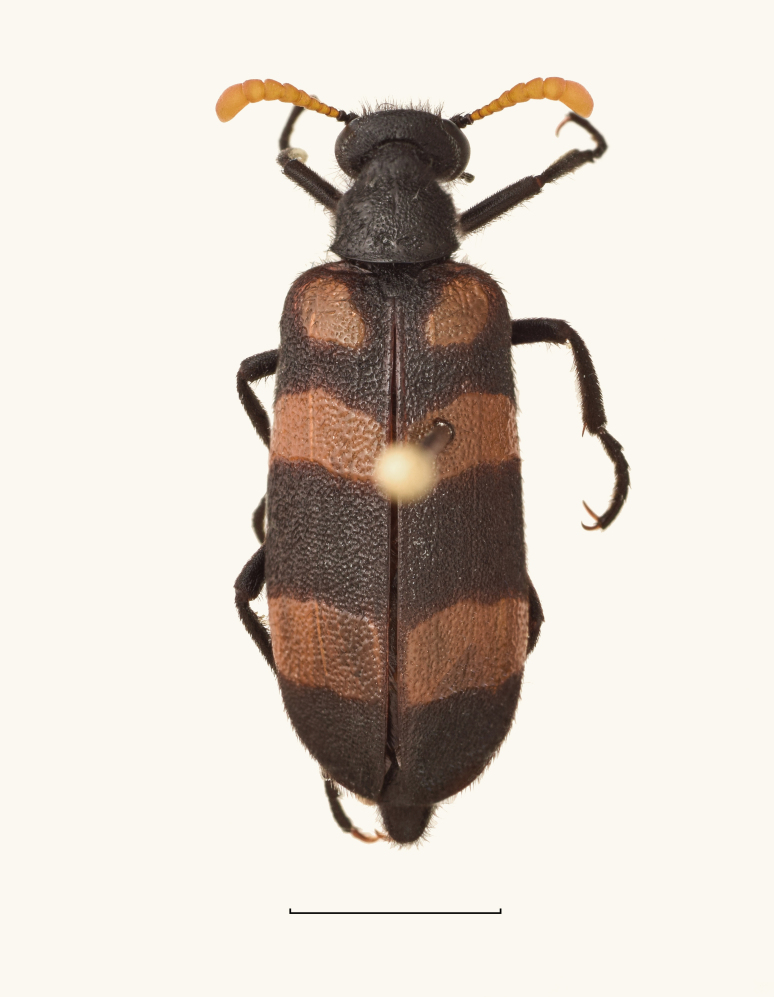
Elytral pattern of *Hycleus scabratus* (VLA_1548), bar size = 5 mm. Photo credits Nikola Vesović.

Most observed specimens of this species represent a typical elytral pattern described by Klug (1834). However, there is also a known form of elytral pattern with a small sub-basal spot which was described by Kaszab (1983) as an aberration beta. The collected specimen has a more expanded sub-basal spot which forms a subtrapezoidal spot. This is the first record of such an elytral pattern (Fig. 4, Suppl. material 13).

#### Hycleus trizonatus

(Reiche, 1866)

97C62B08-CD5F-5590-B059-AEBA68D76ECC

##### Materials

- **Type status:** Other material. **Occurrence:** associatedReferences: MPA_0902; occurrenceID: A5DC9A36-C943-5099-8A79-439B39FCDAA0; **Location:** country: Saudi Arabia; locality: Gharameel; **Event:** eventDate: 7/3/2023

##### Notes

**Figure 5.**
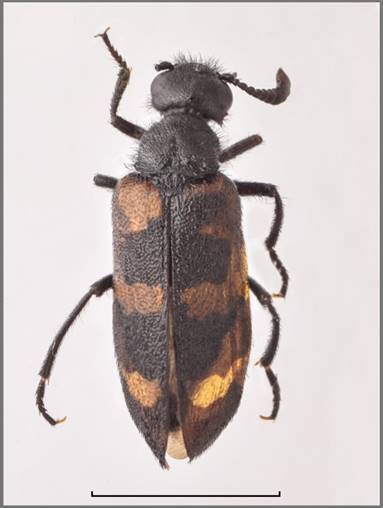
*Hycleus trizonatus* (MPA_0902). Bar size = 5 mm. Photo credits Nikola Vesović.

New record for Saudi Arabia (Fig. 5).

#### Hycleus

2DF70945-3AA9-5485-89E9-288F9A14090C

##### Materials

- **Type status:** Other material. **Occurrence:** associatedReferences: VLA_1520; occurrenceID: BA78E054-5E0F-526F-95D3-9E11F82AE7D3; **Location:** country: Saudi Arabia; locality: Khaybar White Volcano; **Event:** eventDate: 9/5/2023
- **Type status:** Other material. **Occurrence:** associatedReferences: VLA_1626; occurrenceID: B01E4A33-9974-51CD-AD6B-1EB6FA80DF60; **Location:** country: Saudi Arabia; locality: Khaybar White Volcano; **Event:** eventDate: 12/5/2023
- **Type status:** Other material. **Occurrence:** associatedReferences: VLA_2224; occurrenceID: 006C494A-18D9-53A2-ABA6-197504D61E78; **Location:** country: Saudi Arabia; locality: Harrat AlZabin; **Event:** eventDate: 16/6/2023
- **Type status:** Other material. **Occurrence:** associatedReferences: VLA_3057; occurrenceID: 2257719E-92F4-5940-970B-C42BA76675AE; **Location:** country: Saudi Arabia; locality: Jabal Saykhaan; **Event:** eventDate: 12/3/2024
- **Type status:** Other material. **Occurrence:** associatedReferences: VLA_3424; occurrenceID: A7ED4606-6C75-53B4-954C-0699CB08B904; **Location:** country: Saudi Arabia; locality: Triple Bay Mountains; **Event:** eventDate: 12/4/2024
- **Type status:** Other material. **Occurrence:** associatedReferences: VLA_3466; occurrenceID: 52069E0C-98BC-537F-B4C3-B46A1CD17EE5; **Location:** country: Saudi Arabia; locality: Triple Bay Mountains; **Event:** eventDate: 14/4/2024

##### Notes

**Figure 6.**
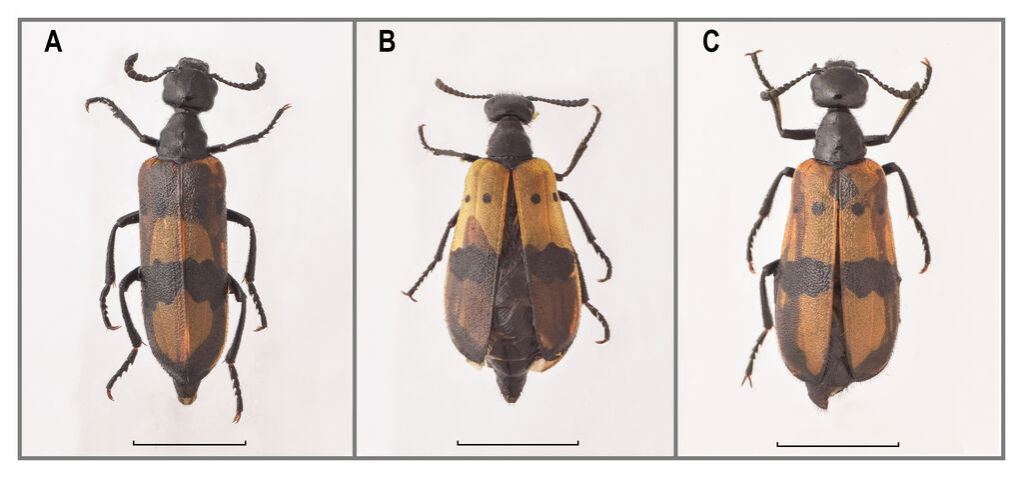
Habitus of *Hycleus* sp.: A) VLA_3424, B) VLA_3466, C) VLA_3057. Bar size = 5 mm. Photocredits Nikola Vesović.

Due to the mesogorbatus type of mesosternum and the shape of male genitalia, this species clearly belongs to the *sexmaculatus* group as defined by Serri et al. (2020). It differs from all known species of the group and it is closest to *H. tenuepictus* known from Anatolia, Egypt and Levant. It has a partly similar elytral pattern, the general shape of the antenna and the last antennal segment. It differs from *H. tenuepictus* due to its slightly reduced ventral blade of the tarsal claws, while, in *H. tenuepictus*, they are well developed and robust. It has a more slender and rather sparsely punctured pronotum. The displayed specimen from Hedjaz in Kaszab (1983) (Plate 2, fig. 21) may also belong to this taxon. Tentatively, it is kept as *Hycleus* sp. (Fig. 6).

#### Mylabris (Eumylabris) calida

(Pallas, 1782)

AD8078C5-125B-54E6-A011-37F5CBBF3712

##### Materials

- **Type status:** Other material. **Occurrence:** associatedReferences: VLA_1490; occurrenceID: BEE53B8C-D94C-5CD1-B44C-20EF5BC75832; **Location:** country: Saudi Arabia; locality: Khaybar White Volcano; **Event:** eventDate: 8/5/2023
- **Type status:** Other material. **Occurrence:** associatedReferences: VLA_1498; occurrenceID: B56E189A-3F17-5FA9-ADA5-1793A257EE7F; **Location:** country: Saudi Arabia; locality: Khaybar White Volcano; **Event:** eventDate: 8/5/2023
- **Type status:** Other material. **Occurrence:** associatedReferences: VLA_1505; occurrenceID: 23837851-AF25-5754-ABA9-00582CED1805; **Location:** country: Saudi Arabia; locality: Khaybar White Volcano; **Event:** eventDate: 9/5/2023
- **Type status:** Other material. **Occurrence:** associatedReferences: VLA_1511; occurrenceID: D715C269-4A07-5BE2-958F-900E4C2DD3E2; **Location:** country: Saudi Arabia; locality: Khaybar White Volcano; **Event:** eventDate: 9/5/2023
- **Type status:** Other material. **Occurrence:** associatedReferences: VLA_1528; occurrenceID: DA8954E3-64BC-51B4-8688-17C9B891587E; **Location:** country: Saudi Arabia; locality: Khaybar White Volcano; **Event:** eventDate: 10/5/2023
- **Type status:** Other material. **Occurrence:** associatedReferences: VLA_1543; occurrenceID: A67770B9-B798-56D8-890D-F8AB04A0A5A1; **Location:** country: Saudi Arabia; locality: Khaybar White Volcano; **Event:** eventDate: 10/5/2023
- **Type status:** Other material. **Occurrence:** associatedReferences: VLA_1605; occurrenceID: 603F32A9-3F41-58DE-95BE-15EB80C30A7D; **Location:** country: Saudi Arabia; locality: Khaybar White Volcano; **Event:** eventDate: 11/5/2023
- **Type status:** Other material. **Occurrence:** associatedReferences: VLA_1620; occurrenceID: 23B2D6B4-2703-5B51-91FA-0F2D006D0CF9; **Location:** country: Saudi Arabia; locality: Khaybar White Volcano; **Event:** eventDate: 12/5/2023

##### Diagnosis

Suppl. material 14.

#### Mylabris (Mylabris) desertica

Bologna, 2007

ADF3C598-752D-5179-BCC9-461971E70C7F

##### Materials

- **Type status:** Other material. **Occurrence:** associatedReferences: VLA_3056; occurrenceID: 5E93DC77-61AC-5DB7-B8B5-124B4F8D28BB; **Location:** country: Saudi Arabia; locality: Jabal Saykhaan; **Event:** eventDate: 12/3/2024
- **Type status:** Other material. **Occurrence:** associatedReferences: VLA_3202; occurrenceID: EA861558-E484-5F64-A65D-739F5BB2603D; **Location:** country: Saudi Arabia; locality: Amaala Northern Mountains; **Event:** eventDate: 21/3/2024
- **Type status:** Other material. **Occurrence:** associatedReferences: VLA_3365; occurrenceID: A6D6458E-561E-570B-AFE3-D0DE1BCD63AE; **Location:** country: Saudi Arabia; locality: Jabal Ral and Northern Areas; **Event:** eventDate: 7/4/2024
- **Type status:** Other material. **Occurrence:** associatedReferences: VLA_3474; occurrenceID: C22D11B5-8C40-5C43-B4FE-D8CC6BEEE424; **Location:** country: Saudi Arabia; locality: Triple Bay Mountains; **Event:** eventDate: 14/4/2024
- **Type status:** Other material. **Occurrence:** associatedReferences: VLA_4104; occurrenceID: 12892F0A-D69E-5860-AF99-24531D1FD98E; **Location:** country: Saudi Arabia; locality: Harrat Lunayyir; **Event:** eventDate: 18/3/2024

#### Mylabris (Mauritabris) filicornis

Marseul, 1870

9DAA18E1-D28C-5729-B404-86469FC21E5A

##### Materials

- **Type status:** Other material. **Occurrence:** associatedReferences: VLA_1622; occurrenceID: DA3A8EAE-B44C-5635-9E72-F666D670EBEF; **Location:** country: Saudi Arabia; locality: Khaybar White Volcano; **Event:** eventDate: 12/5/2023

##### Notes

**Figure 7.**
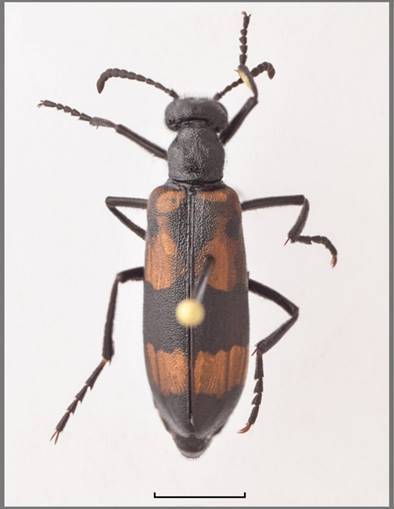
*Mylabris filicornis* (VLA_1622). Bar size = 5 mm. Photo credits Nikola Vesović.

New record for Saudi Arabia (Fig. 7).

##### Diagnosis

Suppl. material 15.

#### Mylabris (Mauritabris) tenebrosa

Laporte de Castelnau, 1840

89FD7EDF-3D82-5085-AF3D-38013866103B

##### Materials

- **Type status:** Other material. **Occurrence:** associatedReferences: VLA_1621; occurrenceID: 44C944B9-EBE6-5C31-B9D9-C77274BA4530; **Location:** country: Saudi Arabia; locality: Khaybar White Volcano; **Event:** eventDate: 12/5/2023
- **Type status:** Other material. **Occurrence:** associatedReferences: VLA_4004; occurrenceID: 8F9DA121-57C0-5FB3-B271-4A1EA8AA9F0B; **Location:** country: Saudi Arabia; locality: Khaybar White Volcano; **Event:** eventDate: 12/5/2023

##### Notes

The first record for the country was published by Schneider (1991). The typical *M. tenebrosa* originally described from Algeria slightly differs. Specimens from Saudi Arabia are larger in body size and have a longer setation of the fore part of the body. These specimens perfectly match the original description of *M. interrupta* (Olivier, 1811) and also partly *M. longipilis* (Pic, 1897), an enigmatic species never studied after its description. Both mentioned species are, according to their description, distributed in Egypt. Further research is needed to clarify the taxonomic status of these species.

##### Diagnosis

Suppl. material 16.

#### Mesomeloe coelatus

Reiche, 1857

43616E0C-8514-593A-807D-08C07EFE16D1

##### Materials

- **Type status:** Other material. **Occurrence:** associatedReferences: BSA_0026; occurrenceID: EFFB4E9D-7B35-5591-9A1C-81C6544E616F; **Location:** country: Saudi Arabia; locality: Jabal Nakhlah; **Event:** eventDate: 2/2/2023
- **Type status:** Other material. **Occurrence:** associatedReferences: VLA_0373; occurrenceID: 429BC809-7C58-574A-8C2E-E814CB80EC3E; **Location:** country: Saudi Arabia; locality: Wadi Nakhlah; **Event:** eventDate: 25/1/2023
- **Type status:** Other material. **Occurrence:** associatedReferences: VLA_0533; occurrenceID: BB4A9541-A4F8-5174-9E30-5A0A505D9AB8; **Location:** country: Saudi Arabia; locality: Jabal Nakhlah; **Event:** eventDate: 2/2/2023
- **Type status:** Other material. **Occurrence:** associatedReferences: VLA_0551; occurrenceID: 99992924-3B53-50DB-99E2-CB8A678B5B95; **Location:** country: Saudi Arabia; locality: Jabal Nakhlah; **Event:** eventDate: 2/2/2023
- **Type status:** Other material. **Occurrence:** associatedReferences: VLA_0890x; occurrenceID: 71330B71-A03E-51D1-9E50-ACD41AD5040E; **Location:** country: Saudi Arabia; locality: Harrat Uwayrid; **Event:** eventDate: 20/2/2023
- **Type status:** Other material. **Occurrence:** associatedReferences: VLA_1212; occurrenceID: D0B3E368-59D3-5D85-BEEE-CD0A30705DBD; **Location:** country: Saudi Arabia; locality: Khaybar White Volcano; **Event:** eventDate: 4/3/2023
- **Type status:** Other material. **Occurrence:** associatedReferences: VLA_1217; occurrenceID: A4E02731-D1BA-5D61-A218-C3CD2D3F2F0F; **Location:** country: Saudi Arabia; locality: Khaybar White Volcano; **Event:** eventDate: 4/3/2023
- **Type status:** Other material. **Occurrence:** associatedReferences: VLA_1228; occurrenceID: C7242FBC-179B-5C99-9E63-B6AD6B442720; **Location:** country: Saudi Arabia; locality: Khaybar White Volcano; **Event:** eventDate: 4/3/2023
- **Type status:** Other material. **Occurrence:** associatedReferences: VLA_3115; occurrenceID: 86AA2887-7FBA-5EBA-817E-F24F063E2254; **Location:** country: Saudi Arabia; locality: Wadi Al Hamd; **Event:** eventDate: 14/3/2024
- **Type status:** Other material. **Occurrence:** associatedReferences: VLA_4003; occurrenceID: DE2CEDDB-0869-57E6-81AB-C5CC4858BFFE; **Location:** country: Saudi Arabia; locality: Jabal Nakhlah; **Event:** eventDate: 3/2/2023

##### Diagnosis

Suppl. material 17.

#### Eurymeloe (Bolognaia) saharensis

(Chobaut, 1898)

258728A5-82B0-5966-B2DA-C2166C6CEC12

##### Materials

- **Type status:** Other material. **Occurrence:** associatedReferences: VLA_0890; occurrenceID: 506D0285-A5DB-5206-B2D3-67DC9ACB9343; **Location:** country: Saudi Arabia; locality: Harrat Uwayrid; **Event:** eventDate: 20/2/2023

##### Diagnosis

Fig. 8.

#### Nemognatha chrysomelina

(Fabricius, 1775)

C1F7D99F-7BC9-5E2E-BFE0-DDF2CDB2DCE5

##### Materials

- **Type status:** Other material. **Occurrence:** associatedReferences: VLA_1518; occurrenceID: B4999358-B492-582F-BC43-952116E6AC19; **Location:** country: Saudi Arabia; locality: Khaybar White Volcano; **Event:** eventDate: 9/5/2023
- **Type status:** Other material. **Occurrence:** associatedReferences: VLA_1616; occurrenceID: A679647F-5F35-5015-95BE-A91D065BCE02; **Location:** country: Saudi Arabia; locality: Khaybar White Volcano; **Event:** eventDate: 11/5/2023

##### Diagnosis

Suppl. material 18.

#### Zonitoschema iranica

Kaszab, 1959

DAFE225A-5A4F-5916-A745-DC13D95F6DA6

##### Materials

- **Type status:** Other material. **Occurrence:** associatedReferences: VLA_2092; occurrenceID: E1A469F8-B5B8-5D7B-BD7B-C606F5B0349E; **Location:** country: Saudi Arabia; locality: Harrat Uwayrid; **Event:** eventDate: 6/6/2023
- **Type status:** Other material. **Occurrence:** associatedReferences: VLA_2100; occurrenceID: 99273815-B16E-589A-B7C4-038D6AD9E590; **Location:** country: Saudi Arabia; locality: Harrat Uwayrid; **Event:** eventDate: 6/6/2023

##### Diagnosis

Suppl. material 19.

#### Zonitoschema rubricolor

Pic, 1924

B5037275-FA6F-5B14-B641-24C5B2ECF913

##### Materials

- **Type status:** Other material. **Occurrence:** associatedReferences: VLA_2282; occurrenceID: 261C711D-E5CD-5665-A6B3-5B983C64F5E8; **Location:** country: Saudi Arabia; locality: Wadi Nakhlah; **Event:** eventDate: 21/6/2023
- **Type status:** Other material. **Occurrence:** associatedReferences: VLA_2305; occurrenceID: B4E8E878-05B9-58A0-94C0-F335A90770FA; **Location:** country: Saudi Arabia; locality: Wadi Nakhlah; **Event:** eventDate: 22/6/2023

##### Diagnosis

Suppl. material 20.

#### Sitaris

DAB870E3-4B24-58C1-A7FB-9877421959CE

##### Materials

- **Type status:** Other material. **Occurrence:** associatedReferences: VLA_3047; occurrenceID: 0A7635D1-81DB-5F05-9FC3-31DF49246BE3; **Location:** country: Saudi Arabia; locality: Harrat Uwayrid; **Event:** eventDate: 5/6/2023

##### Notes

**Figure 9.**
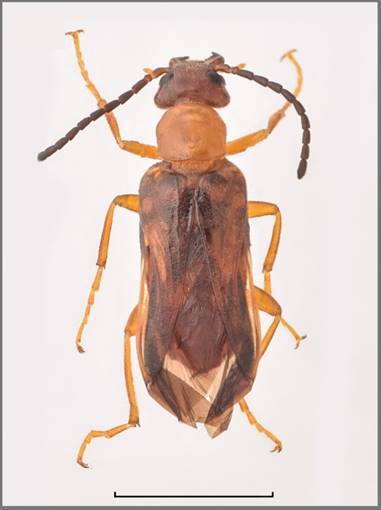
*Sitaris* sp. (VLA_3047). Bar size 5 mm. Photo credis Nikola Vesović.

This is the first record of this genus for the Saudi Arabia, but, as the genus has not been studied recently, the identification to the species level is currently not possible (Fig. 9).

## Analysis

### Distribution

**Figure 10.**
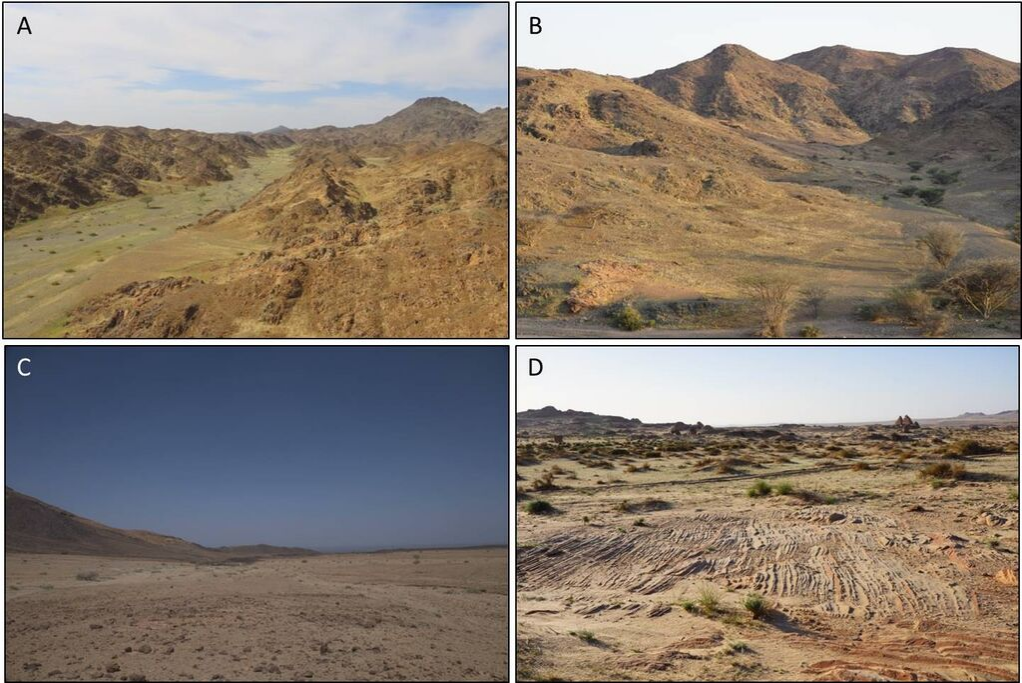
Studied habitats. **A, B** sites of *M. desertica* in Jabal Ral and Triple Bay Mountains, respectively; **C** site of *H. borchmannianus* and *M. filicornis* in Khaybar White Volcano Geopark; **D** site of *H. trizonatus* in Gharameel.

Preliminary distribution patterns were found amongst the recorded meloid beetles (Suppl. material 21). In the study area, *L. angusticollis suturellus* was found in slopes of low-altitude mountains along the Red Sea coast, *L. brittoni* was apparently restricted to high-altitude rocky habitats in the Khaybar volcanic area, while *L. palaestinus* was found only in sandy habitats interspersed with sandstone outcrops. Apparently, there was clear habitat segregation between *Zonitoschema* species in the study area, where *Z. iranica* was observed in rocky habitats of Harrat Uwayrid, while *Z. rubricolor* was found in sandy areas characterised by well-developed vegetation. In the study area, specimens of *M. desertica* were observed in lowland areas along the Red Sea coast, where it occupies rocky habitats with sparse vegetation. *M. coelatus* was relatively widespread in the region, recorded from five protected areas. However, *E. saharensis* was found only at a single high-altitude site, where it co-occurred with *M. coelatus*. Two newly-recorded species for the country, *H. borchmannianus* and *M. filicornis*, were also found in high-altitude areas of the Khaybar volcanoes. In contrast, *H. trizonatus* was found in sandy areas with abundant rock formations in the Gharameel region. In one of the most remote parts of Harrat Uwayrid, a single specimen of *Sitaris* was discovered in a small wadi formed along lava flow paths (Fig. 10).

**Figure 11.**
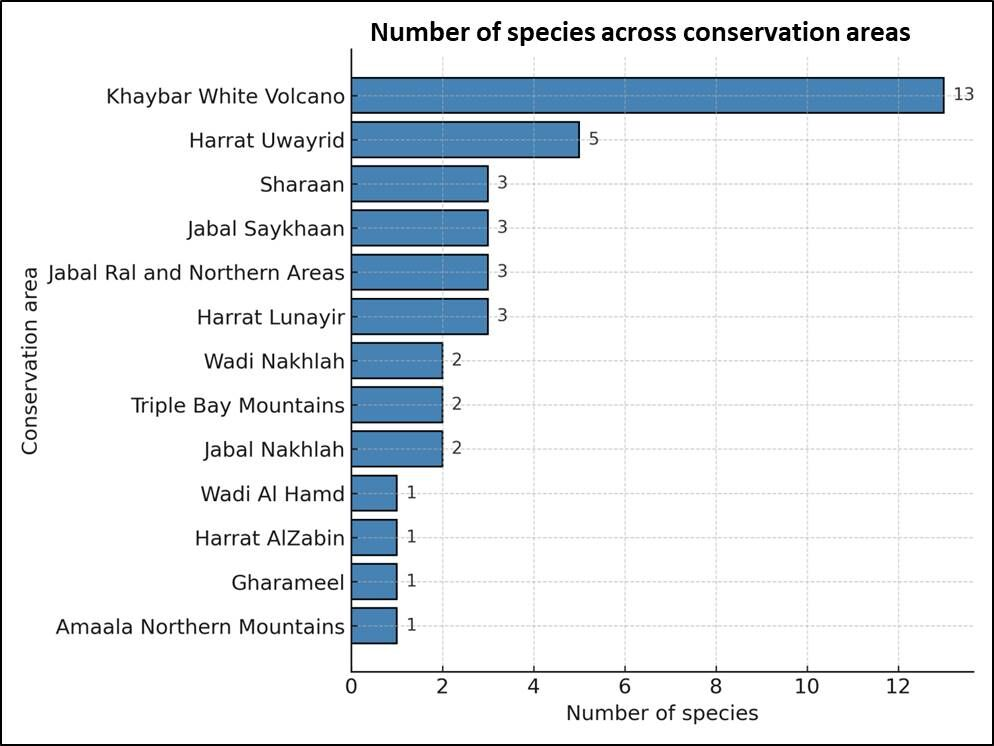
Number of species recorded across conservation areas.

The area of Khaybar White Volcano Geopark was particularly rich in meloid diversity. Of a total of 24 recorded taxa for the study area, 13 were present in this protected area. Besides that, of those 13 taxa, nine were found exclusively there (Fig. 11). This area is highly remote with low accessibility roads, hence it is one of the most well-conserved places in the study area. The area is represented by rocks, lava fields and a significant number of inactive volcanoes. It is rich in *wadis* formed in lava flows, which support a high abundance and diversity of vegetation (Suppl. material 22).

## Discussion

In this study, a total of 21 species and three subspecies belonging to 11 genera of the family Meloidae were recorded from north-western Saudi Arabia. Amongst those, three species (*Hycleus borchmannianus*, *Hycleus trizonatus*, *Mylabris filicornis*) and one genus (*Sitaris*) are recorded for the first time in Saudi Arabia. With those discoveries, the number of recorded meloid beetles of Saudi Arabia totals 88.

High-altitude volcanic habitats, particularly in the Khaybar region, emerged as hotspots of meloid diversity in the study area, with several species restricted to this unique landscape, including the two newly-recorded taxa for the country. The richness of the Khaybar White Volcano Geopark underscores the role of remote and relatively undisturbed habitats in supporting specialised beetle assemblages, while the discovery of rare taxa, such as *Sitaris* in isolated *wadis* further emphasises the importance of microhabitats formed by lava flows. In contrast, species, such as *M. coelatus*, exhibited broader ecological tolerance, occurring across multiple protected areas, whereas others, like *E. saharensis*, appeared highly restricted. Although, due to its cryptic lifestyle and winter activity, the apparently restricted distribution of *E. saharensis* we observed in the study area is likely a consequence of insufficient sampling, especially considering its known broad range (Ruiz et al. 2010, Sánchez Vialas et al. 2024). Together, these findings suggest that the mosaic of sandy plains, rocky outcrops and volcanic landscapes in the region creates distinct ecological niches that sustain a diverse meloid fauna, reinforcing the conservation value of these geologically and biologically unique habitats.

Overall, this study highlights that the north-western region of Saudi Arabia remains underexplored in terms of Meloidae diversity. As shown by existing literature and confirmed again by our results, the surveys in this region have led to the discovery of species or subspecies either new to the country or new to science (e.g. Licata et al. (2024)). This strongly suggests that additional fieldwork in these remote and diverse habitats will yield further significant findings and contribute to a more comprehensive understanding of biodiversity in Saudi Arabia.

## Supplementary Material

XML Treatment for Mylabris (Mylabris) desertica

XML Treatment for Alosimus syriacus syriacus

XML Treatment for Lydomorphus angusticollis suturellus

XML Treatment for Lydomorphus brittoni

XML Treatment for Lydomorphus palaestinus

XML Treatment for Ammabris elegans

XML Treatment for Croscherichia goryi

XML Treatment for Croscherichia sanguinolenta arabica

XML Treatment for Croscherichia tigrinipennis

XML Treatment for Hycleus borchmannianus

XML Treatment for Hycleus novemdecimpunctatus

XML Treatment for Hycleus pseudobrunnipes

XML Treatment for Hycleus scabratus

XML Treatment for Hycleus trizonatus

XML Treatment for Hycleus

XML Treatment for Mylabris (Eumylabris) calida

XML Treatment for Mylabris (Mylabris) desertica

XML Treatment for Mylabris (Mauritabris) filicornis

XML Treatment for Mylabris (Mauritabris) tenebrosa

XML Treatment for Mesomeloe coelatus

XML Treatment for Eurymeloe (Bolognaia) saharensis

XML Treatment for Nemognatha chrysomelina

XML Treatment for Zonitoschema iranica

XML Treatment for Zonitoschema rubricolor

XML Treatment for Sitaris

80CE8358-F2C4-5045-B643-7BDA93AF6207 10.3897/BDJ.13.e174504.suppl1 Supplementary material 1 Complete Meloidae dataset Data type table File: oo_1484540.xlsx https://binary.pensoft.net/file/1484540 Vidak Lakušić

BCE2DC62-10A9-521A-B8A1-D6B57B9A0913 10.3897/BDJ.13.e174504.suppl2 Supplementary material 2 Alosimus syriacus VLA_0852 Data type Image File: oo_1407089.JPG https://binary.pensoft.net/file/1407089 Vidak Lakušić

54CD3580-5F2E-510B-AD04-F99B63ED5E23 10.3897/BDJ.13.e174504.suppl3 Supplementary material 3 Lydomorphus angusticollis suturellus (VLA_3325) Data type Image File: oo_1406222.JPG https://binary.pensoft.net/file/1406222 Vidak Lakušić

E890817D-D142-5215-9CFD-28FD20EEC77B 10.3897/BDJ.13.e174504.suppl4 Supplementary material 4 Lydomorphus brittoni (VLA_1614) Data type Image File: oo_1412179.JPG https://binary.pensoft.net/file/1412179 Vidak Lakušić

ABABDD87-01AA-51D3-BCC3-C5CC56ACC29D 10.3897/BDJ.13.e174504.suppl5 Supplementary material 5 Lydomorphus palaestinus (VLA_0994) Data type Image File: oo_1406225.JPG https://binary.pensoft.net/file/1406225 Vidak Lakušić

6F250074-A3E5-53F4-94F3-85B533BB3BB4 10.3897/BDJ.13.e174504.suppl6 Supplementary material 6 Ammabris elegans (VLA_1812) Data type Image File: oo_1412181.JPG https://binary.pensoft.net/file/1412181 Vidak Lakušić

A14DC6F3-E3C4-56D2-9F19-4FE166A377A6 10.3897/BDJ.13.e174504.suppl7 Supplementary material 7 Croscherichia goryi (VLA_1868) Data type Image File: oo_1412182.JPG https://binary.pensoft.net/file/1412182 Vidak Lakušić

EB5694C9-37E6-5ED9-A153-E4DE48ED5870 10.3897/BDJ.13.e174504.suppl8 Supplementary material 8 Croscherichia sanguinolenta arabica (VLA_1542) Data type Image File: oo_1406229.JPG https://binary.pensoft.net/file/1406229 Vidak Lakušić

1913A800-55EA-5420-8A2F-217F413F4D9B 10.3897/BDJ.13.e174504.suppl9 Supplementary material 9 Croscherichia tigrinipennis (VLA_3125) Data type Image File: oo_1412198.JPG https://binary.pensoft.net/file/1412198 Vidak Lakušić

7EE3C7E3-BD46-583C-9A56-92A48202209B 10.3897/BDJ.13.e174504.suppl10 Supplementary material 10 Hycleus borchmannianus (VLA_1627) Data type Image File: oo_1406245.JPG https://binary.pensoft.net/file/1406245 Vidak Lakušić

07F4F4A7-848F-51F9-B396-62BDE11995C9 10.3897/BDJ.13.e174504.suppl11 Supplementary material 11 Hycleus novemdecimpunctatus (VLA_1624) Data type Image File: oo_1406233.JPG https://binary.pensoft.net/file/1406233 Vidak Lakušić

B3926A5E-9D15-564C-8F0F-D22206F23631 10.3897/BDJ.13.e174504.suppl12 Supplementary material 12 Hycleus pseudobrunnipes (VLA_1512) Data type Image File: oo_1407098.JPG https://binary.pensoft.net/file/1407098 Vidak Lakušić

BC0868B2-7B31-5215-8CF9-2C6CE435C76A 10.3897/BDJ.13.e174504.suppl13 Supplementary material 13 Hycleus scabratus (VLA_1548) Data type Image File: oo_1406246.JPG https://binary.pensoft.net/file/1406246 Vidak Lakušić

0B00F242-4804-5074-90EA-09CA600CC1F2 10.3897/BDJ.13.e174504.suppl14 Supplementary material 14 Mylabris calida (VLA_1505) Data type Image File: oo_1412187.JPG https://binary.pensoft.net/file/1412187 Vidak Lakušić

7FABEA00-6861-54B5-982A-2FB2BA38B931 10.3897/BDJ.13.e174504.suppl15 Supplementary material 15 Mylabris filicornis (VLA_1622) Data type Image File: oo_1406247.JPG https://binary.pensoft.net/file/1406247 Vidak Lakušić

6363A956-E53A-5EB6-98EE-063C02EBCB6F 10.3897/BDJ.13.e174504.suppl16 Supplementary material 16 Mylabris tenebrosa (VLA_1621) Data type Image File: oo_1406238.JPG https://binary.pensoft.net/file/1406238 Vidak Lakušić

3F8BD2C9-CB4E-560C-8C0C-B350DF7707B3 10.3897/BDJ.13.e174504.suppl17 Supplementary material 17 Mesomeloe coelatus (VLA_0373) Data type Image File: oo_1406239.JPG https://binary.pensoft.net/file/1406239 Vidak Lakušić

84D64313-1E12-532C-B4EC-A2AE2915AF85 10.3897/BDJ.13.e174504.suppl18 Supplementary material 18 Nemognatha chrysomelina (VLA_1518) Data type Image File: oo_1412199.JPG https://binary.pensoft.net/file/1412199 Vidak Lakušić

135726B6-0DD4-5C46-9709-177DB53A0FD7 10.3897/BDJ.13.e174504.suppl19 Supplementary material 19 Zonitoschema iranica (VLA_2092) Data type Image File: oo_1412193.JPG https://binary.pensoft.net/file/1412193 Vidak Lakušić

8AE51C5A-4F60-5E48-91C3-9341A5D7E9E2 10.3897/BDJ.13.e174504.suppl20 Supplementary material 20 Zonitoschema rubricolor (VLA_2282) Data type Image File: oo_1406244.JPG https://binary.pensoft.net/file/1406244 Vidak Lakušić

600281A9-468E-57DD-94D9-B52D467C207F 10.3897/BDJ.13.e174504.suppl21 Supplementary material 21 Meloidae species distribution in the study area Data type image File: oo_1489935.jpg https://binary.pensoft.net/file/1489935 Vidak Lakušić

7BAE22DC-2714-5A45-81A6-8B4563FD6F0B 10.3897/BDJ.13.e174504.suppl22 Supplementary material 22 Sites in Khaybar White Volcano Geopark. Data type Image File: oo_1410279.jpg https://binary.pensoft.net/file/1410279 BIOPOLIS

## Figures and Tables

**Figure 8.**
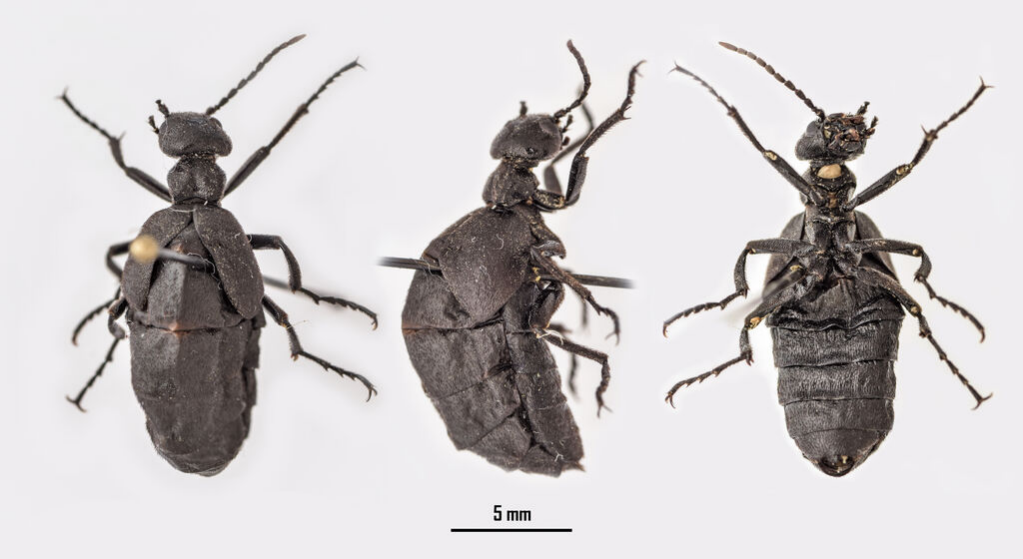
*Eurymeloe saharensis* (VLA_0890). Photo credits Nikola Vesović.
