# Supplementary figures and images for: Diversity and distribution of blister beetles (Coleoptera, Meloidae) from north-western Saudi Arabia: new observations and first description of a male Mylabris desertica Bologna, 2007
Source: Biodivers Data J. 2025 Dec 22;13:e174504. doi: 10.3897/BDJ.13.e174504 (PMC12750105; doi:10.3897/BDJ.13.e174504)

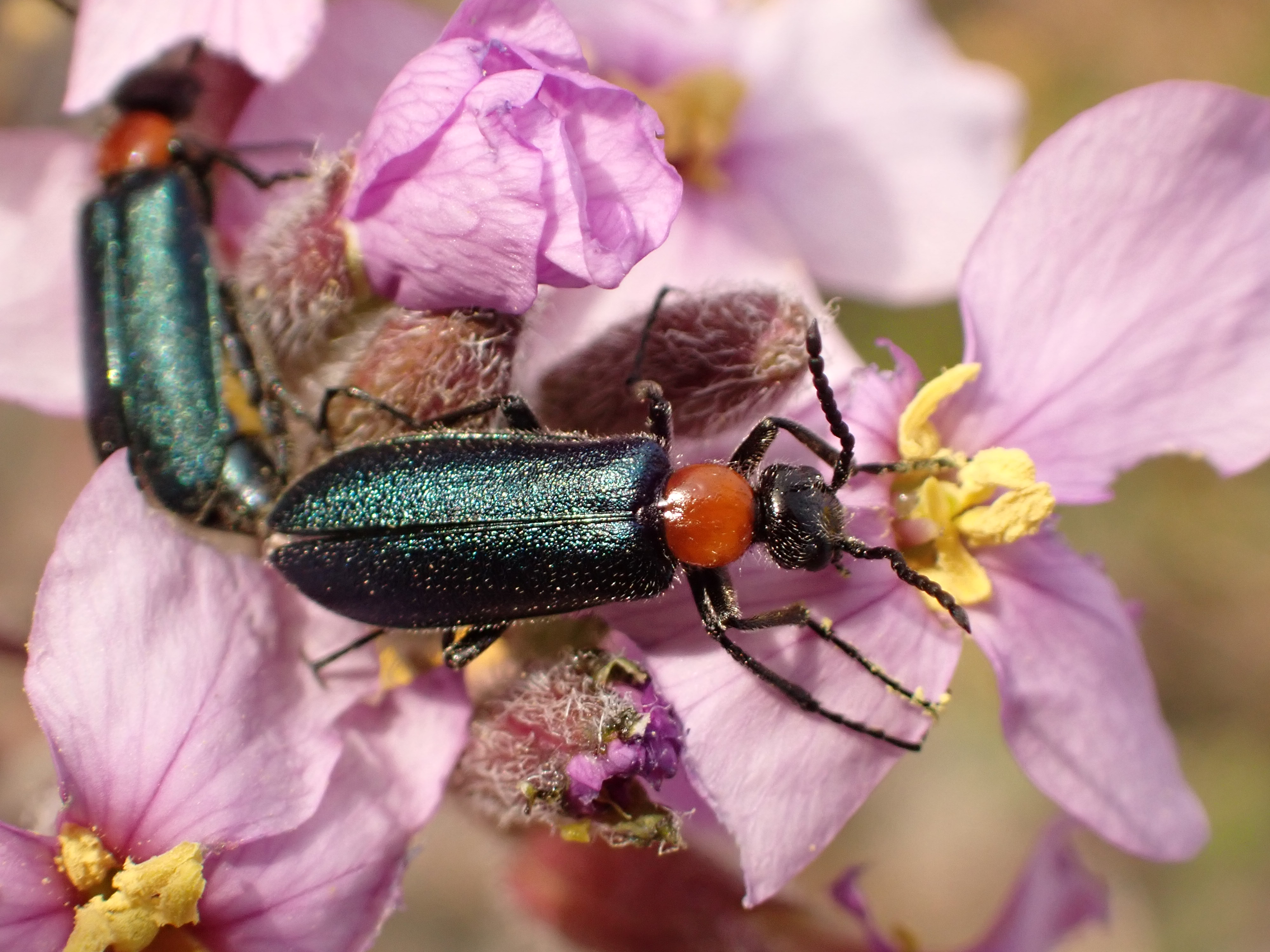

Supplement: Supplementary material 2 — Alosimus syriacus VLA_0852 [file bdj-13-e174504-s002.jpg]

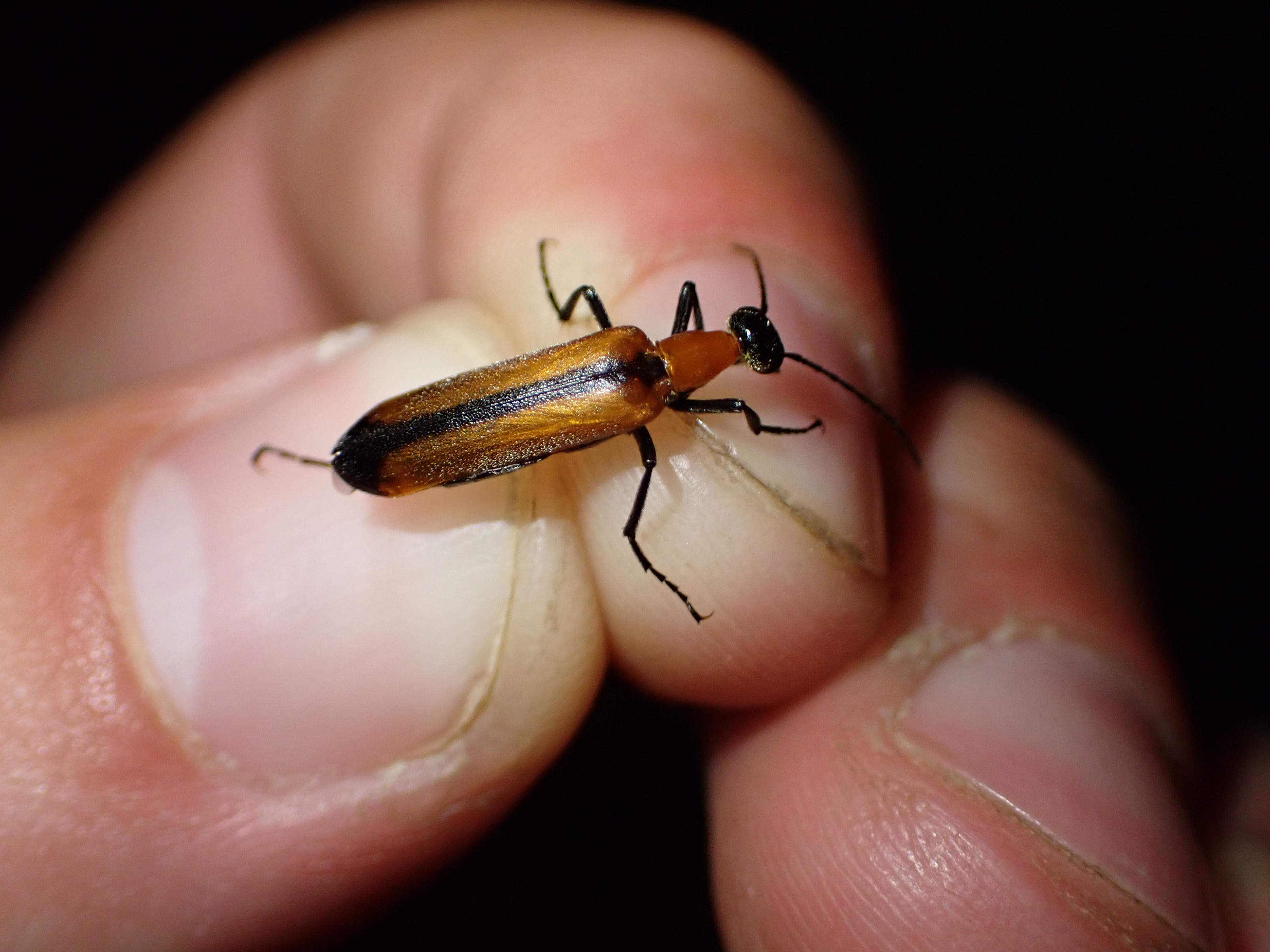

Supplement: Supplementary material 3 — Lydomorphus angusticollis suturellus (VLA_3325) [file bdj-13-e174504-s003.jpg]

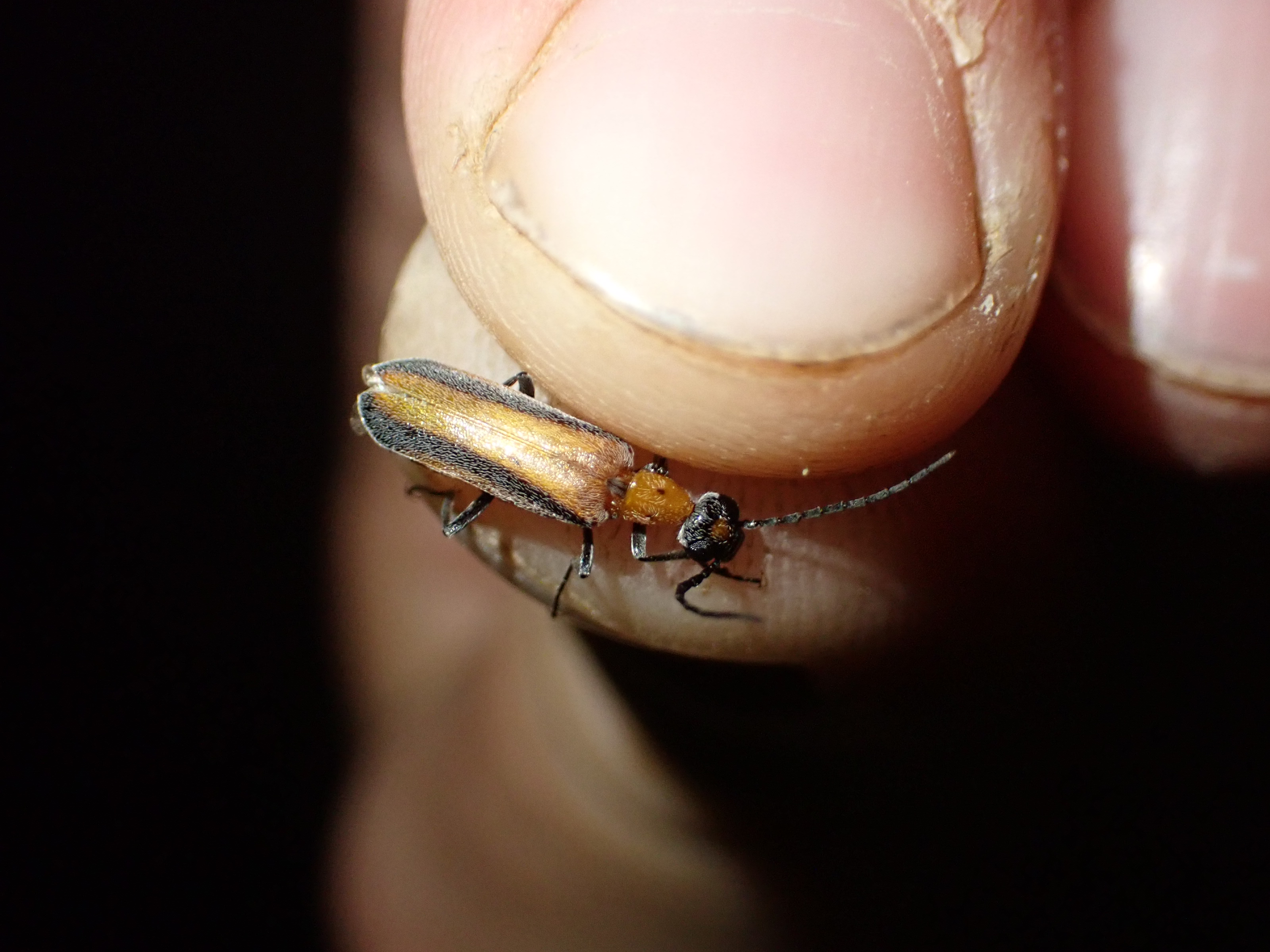

Supplement: Supplementary material 4 — Lydomorphus brittoni (VLA_1614) [file bdj-13-e174504-s004.jpg]

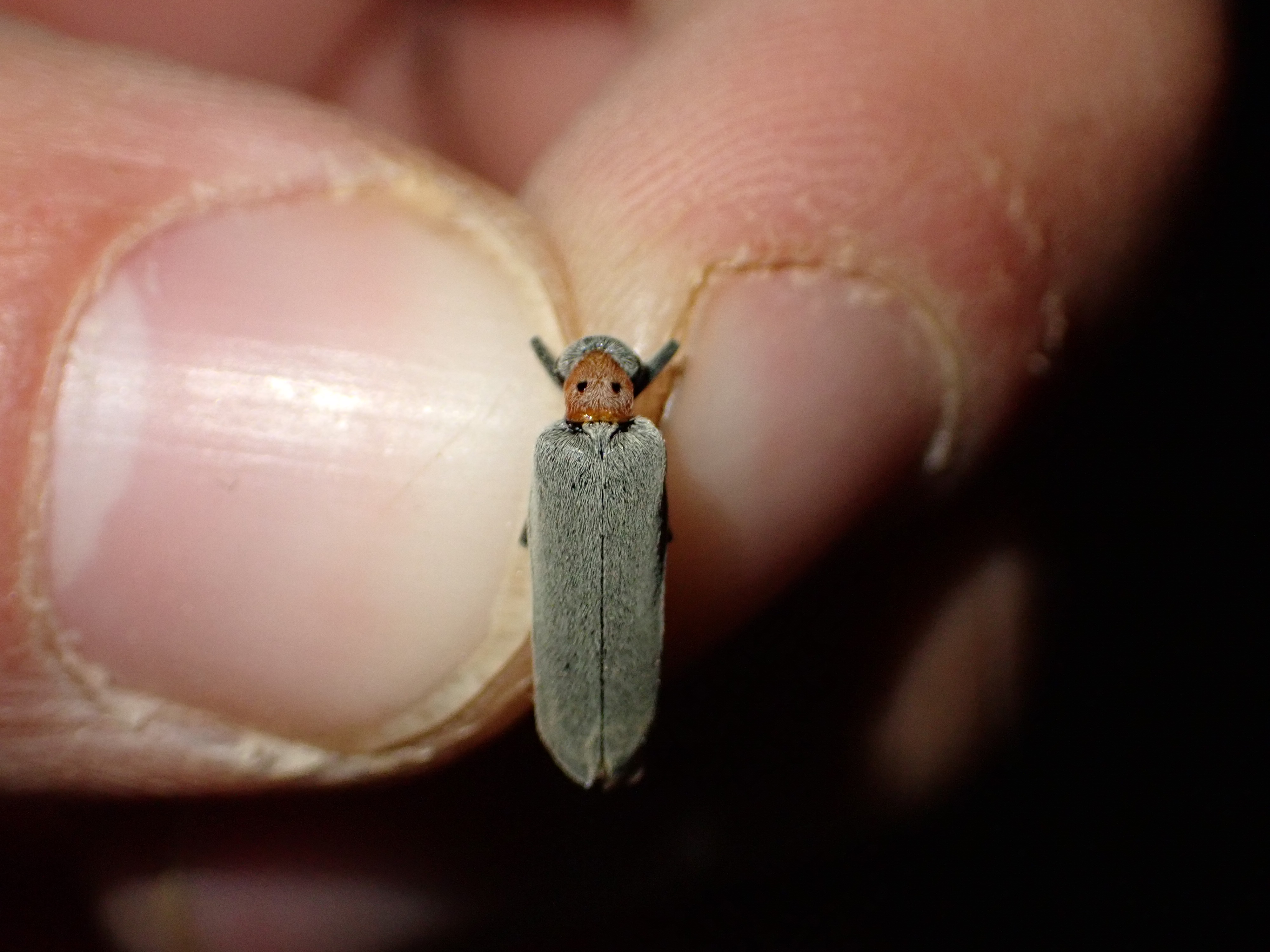

Supplement: Supplementary material 5 — Lydomorphus palaestinus (VLA_0994) [file bdj-13-e174504-s005.jpg]

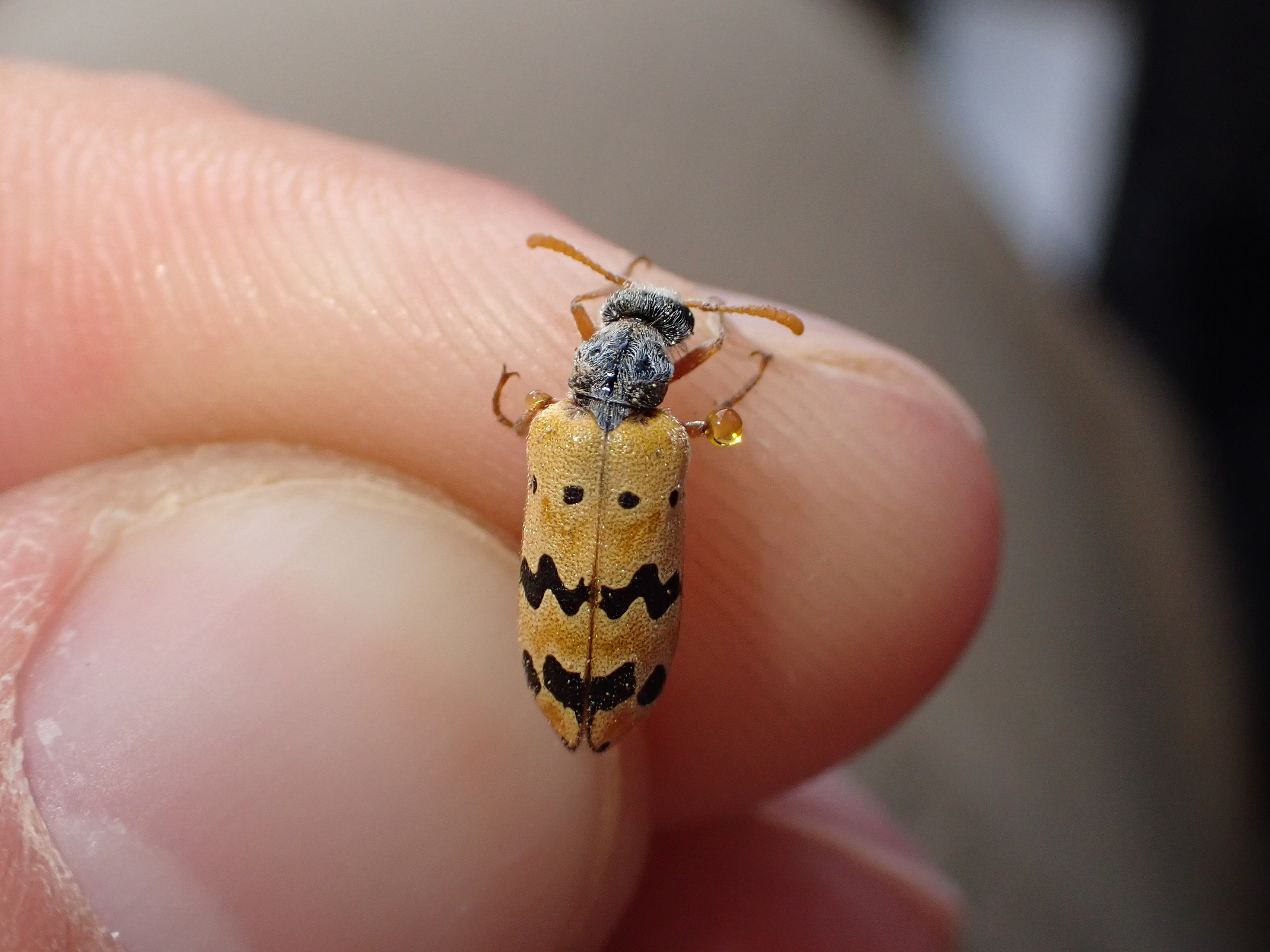

Supplement: Supplementary material 6 — Ammabris elegans (VLA_1812) [file bdj-13-e174504-s006.jpg]

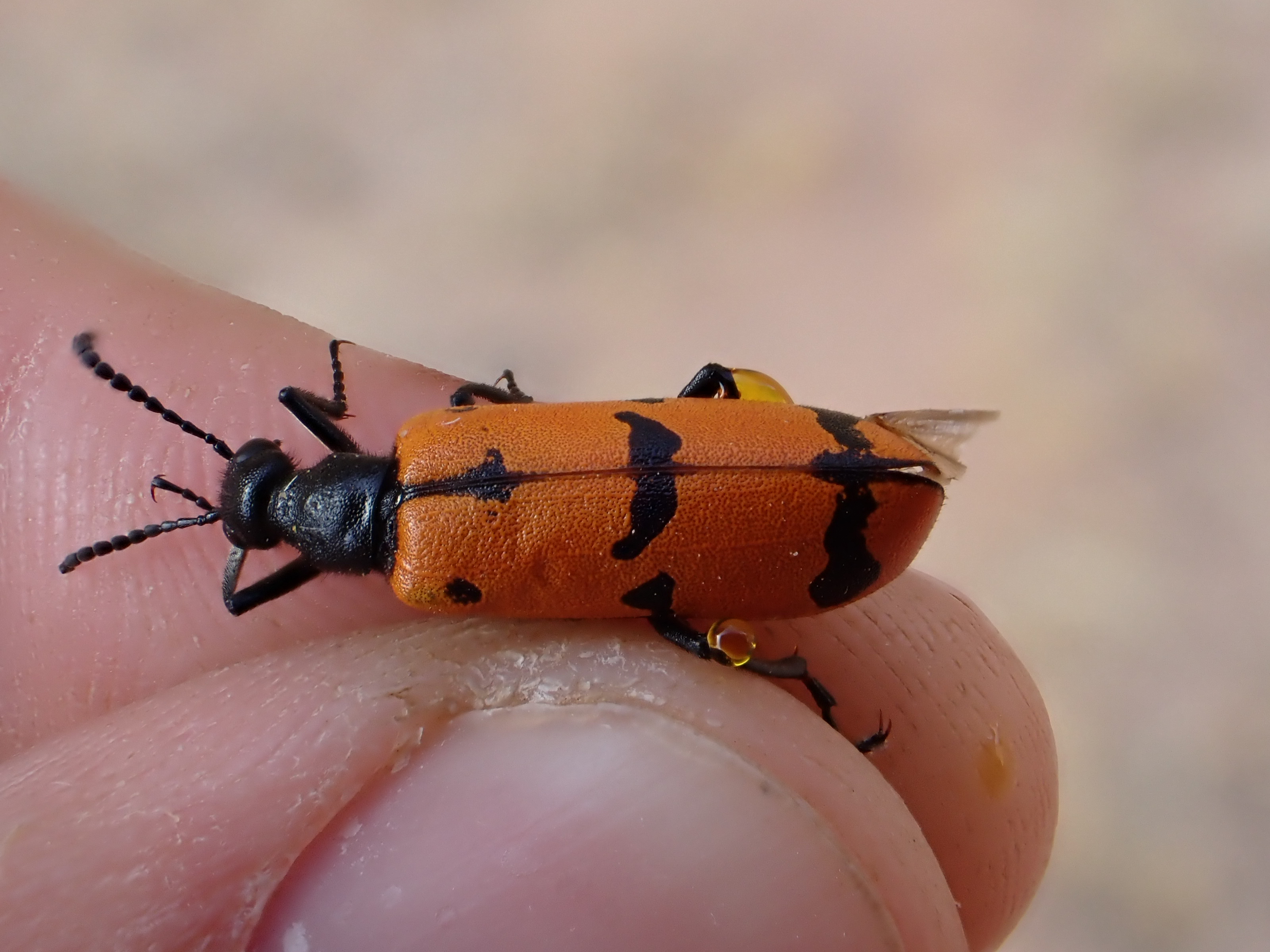

Supplement: Supplementary material 7 — Croscherichia goryi (VLA_1868) [file bdj-13-e174504-s007.jpg]

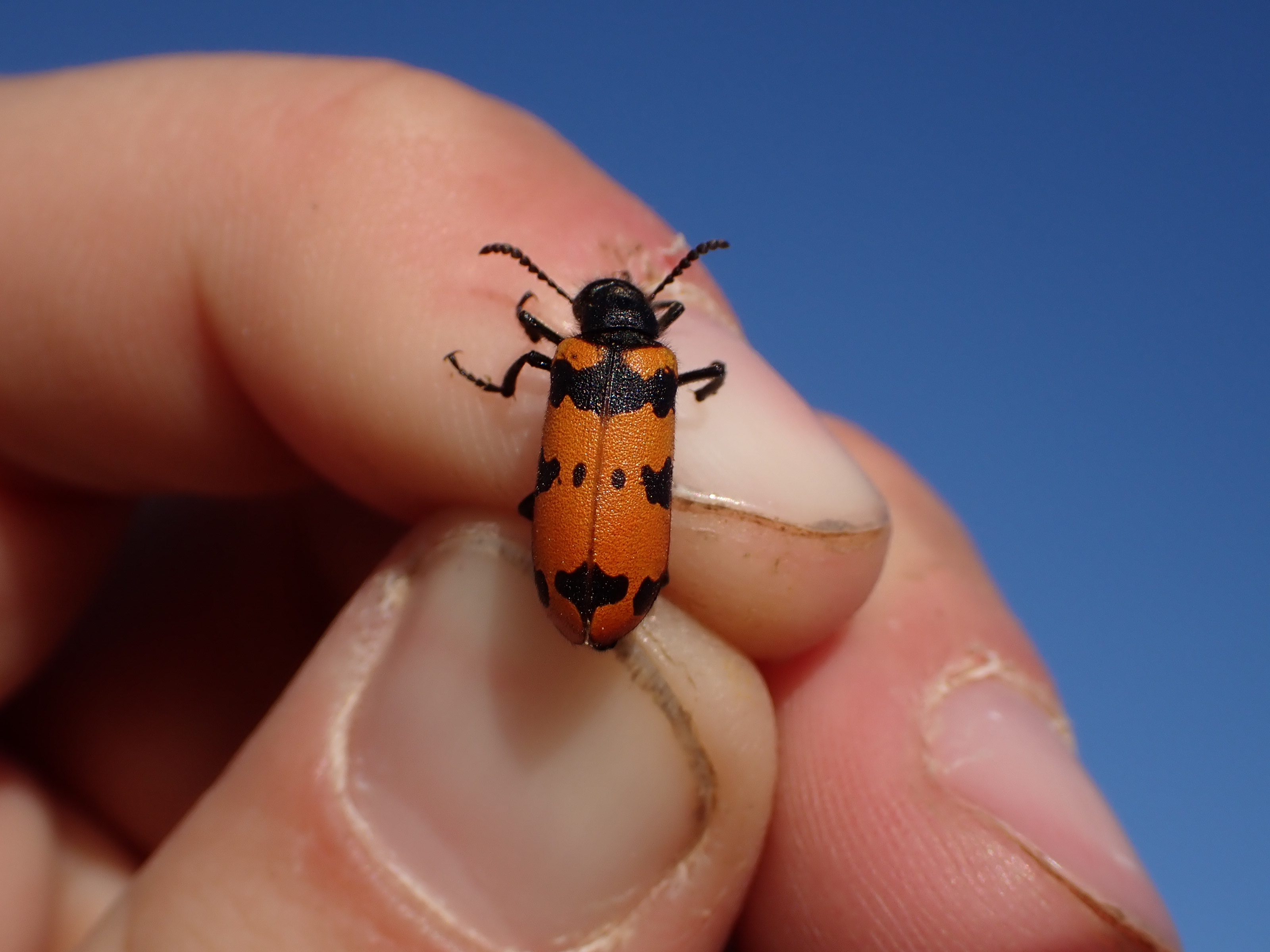

Supplement: Supplementary material 8 — Croscherichia sanguinolenta arabica (VLA_1542) [file bdj-13-e174504-s008.jpg]

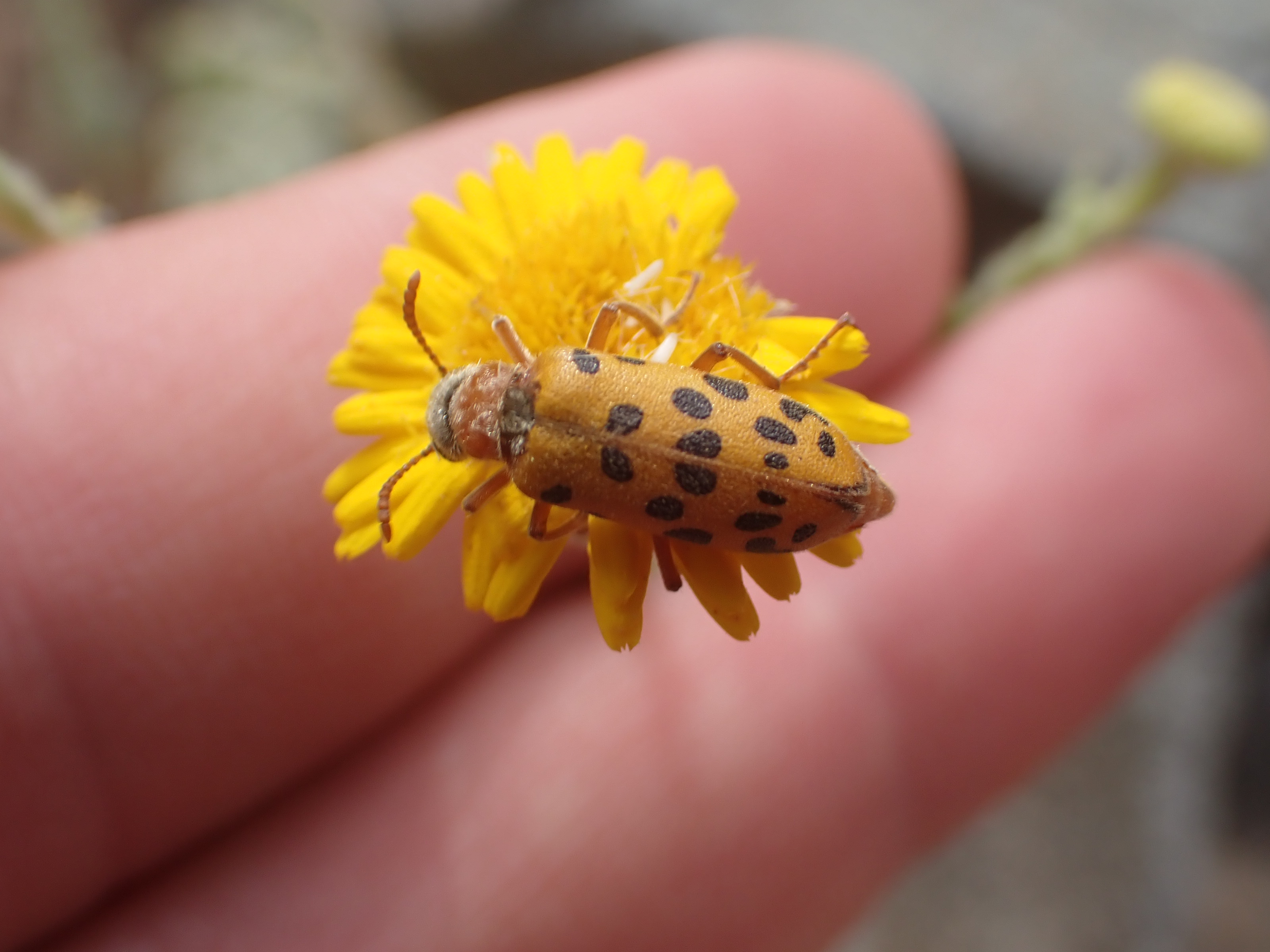

Supplement: Supplementary material 9 — Croscherichia tigrinipennis (VLA_3125) [file bdj-13-e174504-s009.jpg]

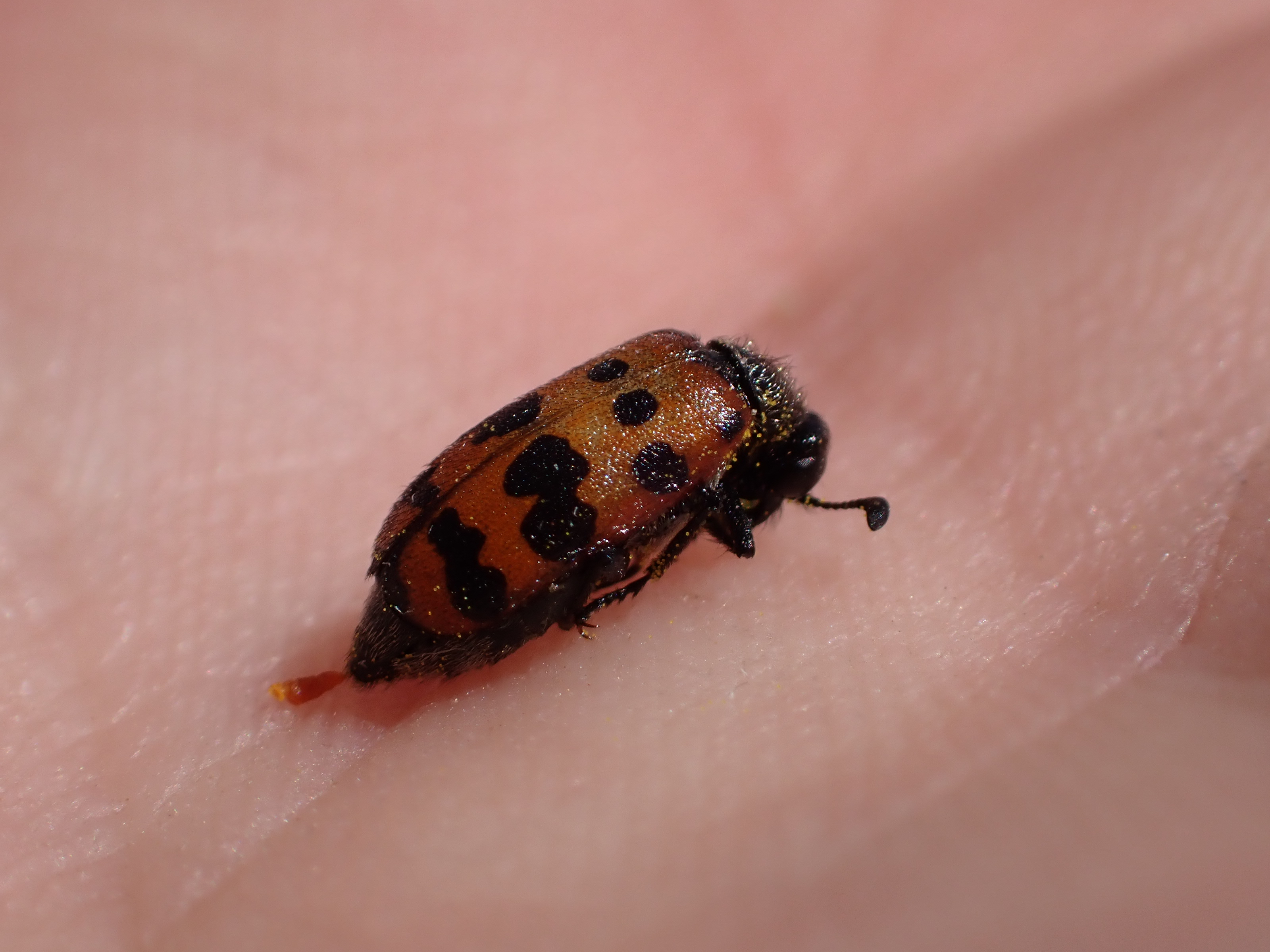

Supplement: Supplementary material 10 — Hycleus borchmannianus (VLA_1627) [file bdj-13-e174504-s010.jpg]

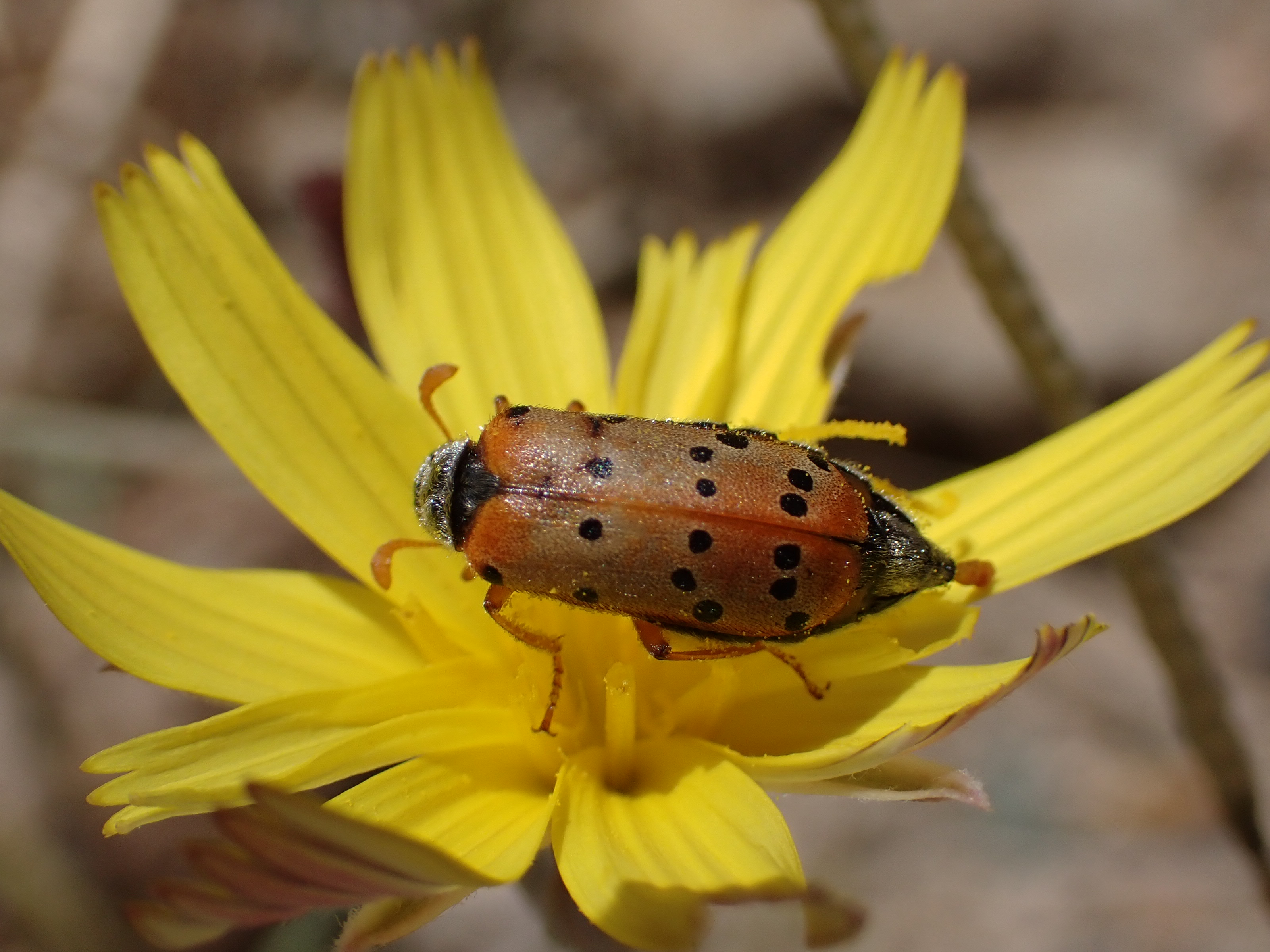

Supplement: Supplementary material 11 — Hycleus novemdecimpunctatus (VLA_1624) [file bdj-13-e174504-s011.jpg]

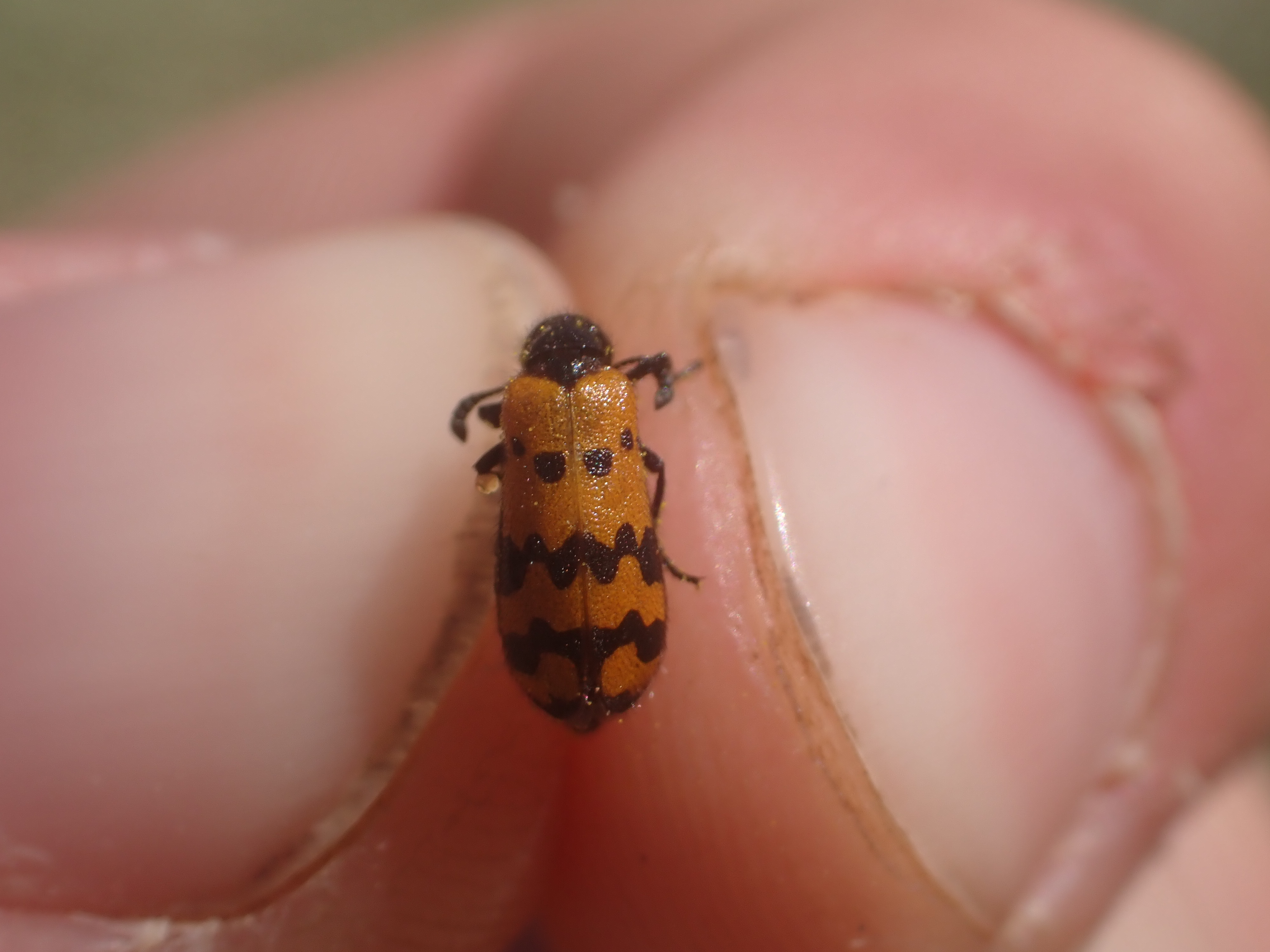

Supplement: Supplementary material 12 — Hycleus pseudobrunnipes (VLA_1512) [file bdj-13-e174504-s012.jpg]

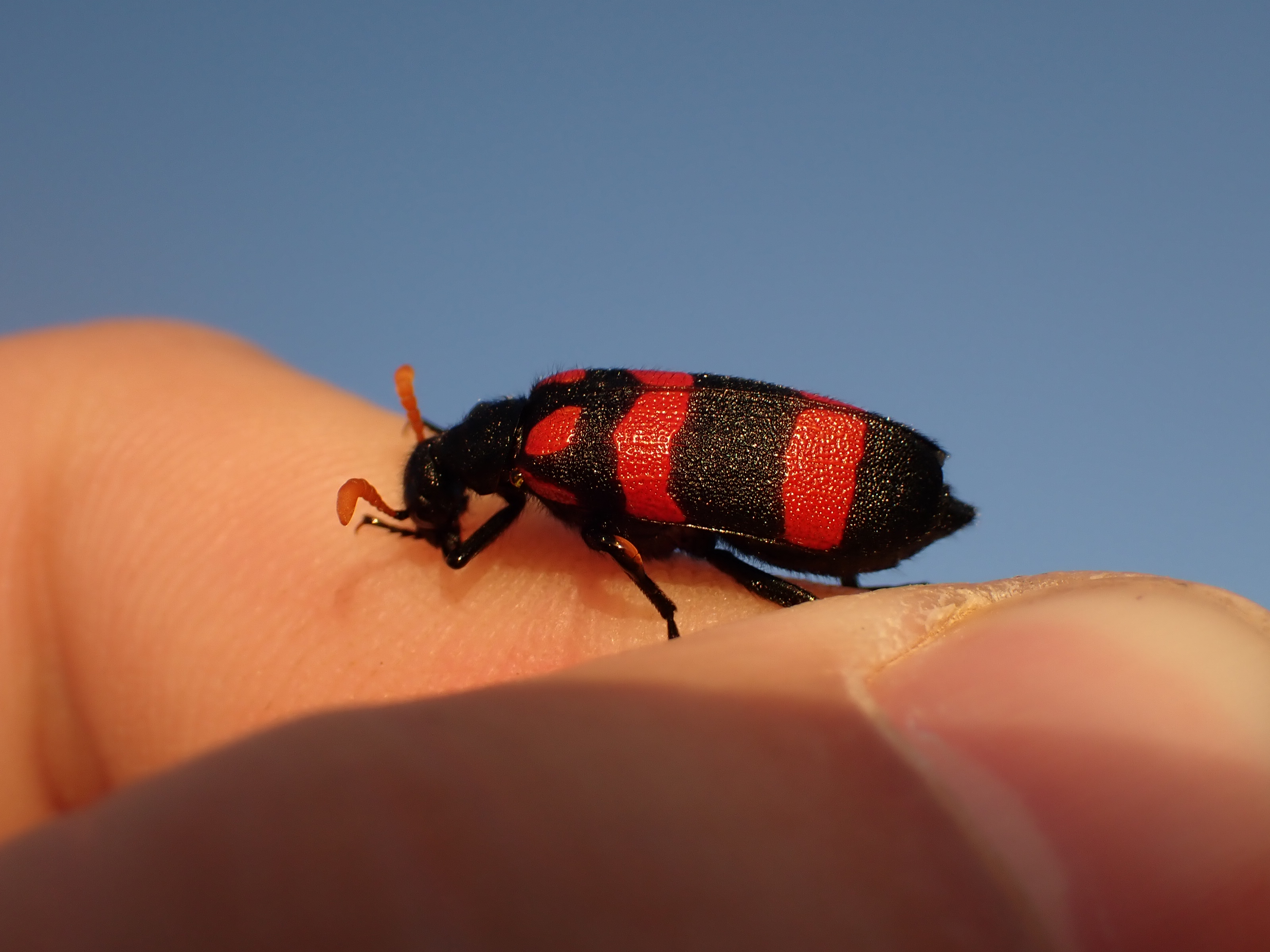

Supplement: Supplementary material 13 — Hycleus scabratus (VLA_1548) [file bdj-13-e174504-s013.jpg]

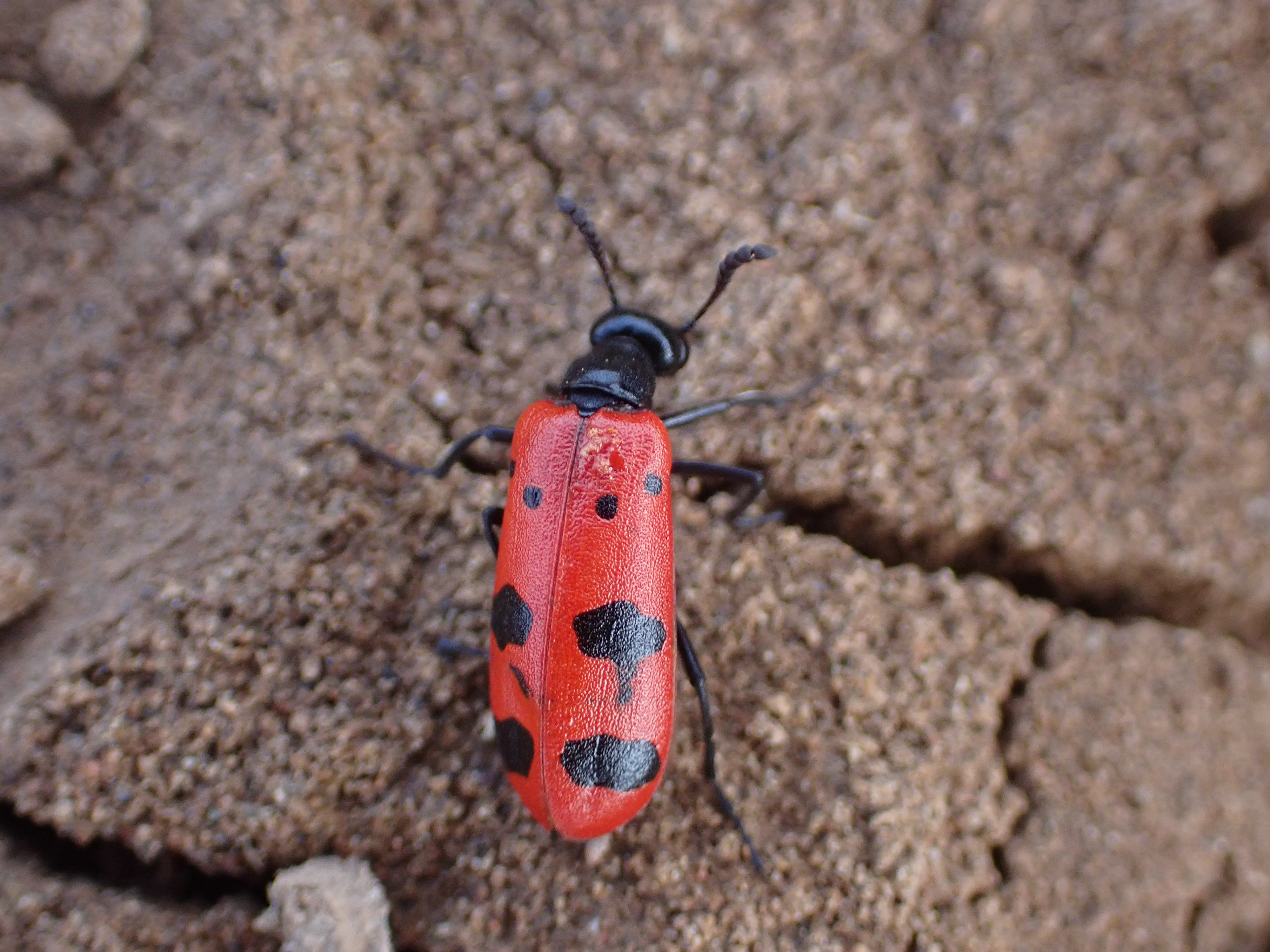

Supplement: Supplementary material 14 — Mylabris calida (VLA_1505) [file bdj-13-e174504-s014.jpg]

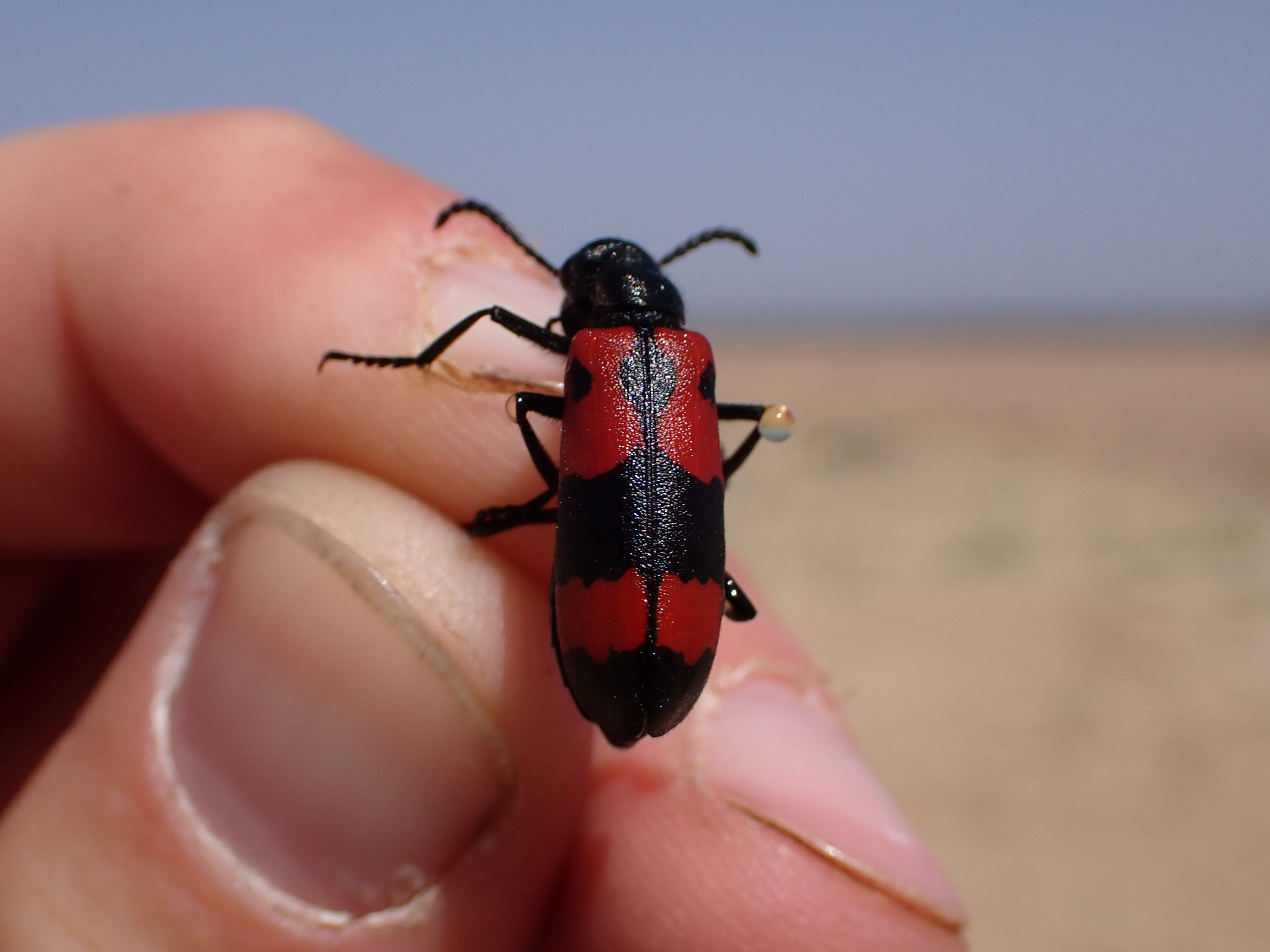

Supplement: Supplementary material 15 — Mylabris filicornis (VLA_1622) [file bdj-13-e174504-s015.jpg]

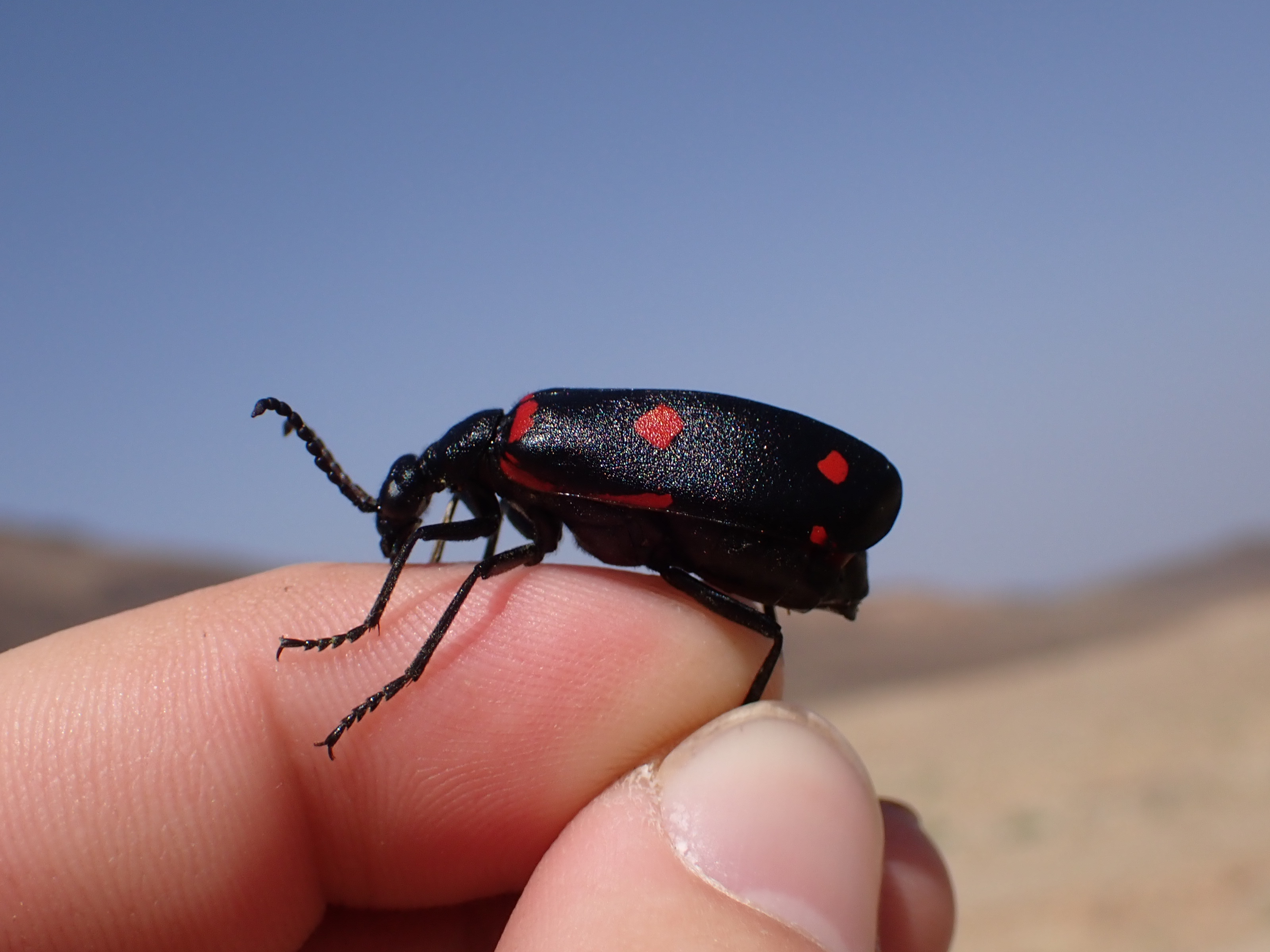

Supplement: Supplementary material 16 — Mylabris tenebrosa (VLA_1621) [file bdj-13-e174504-s016.jpg]

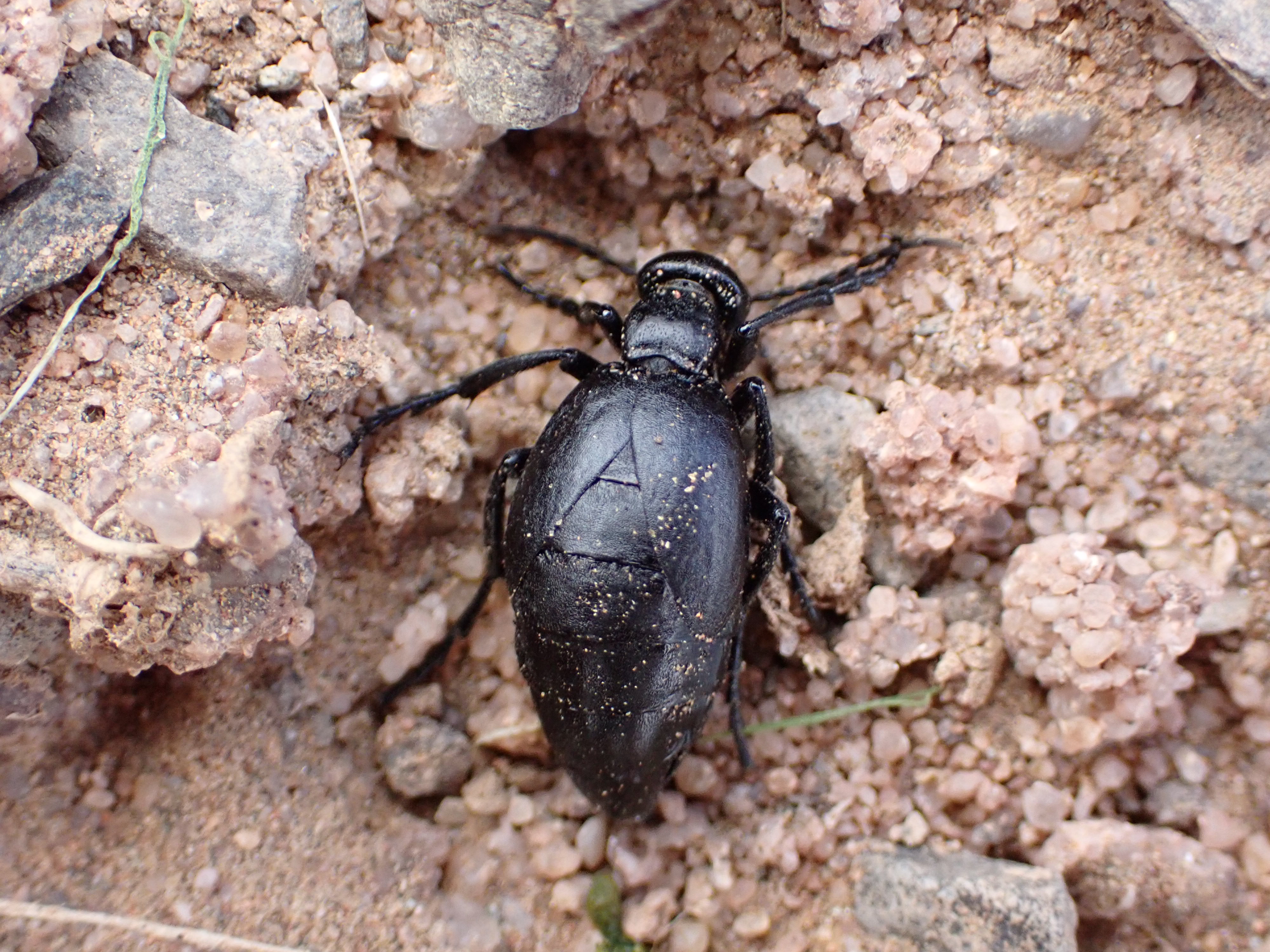

Supplement: Supplementary material 17 — Mesomeloe coelatus (VLA_0373) [file bdj-13-e174504-s017.jpg]

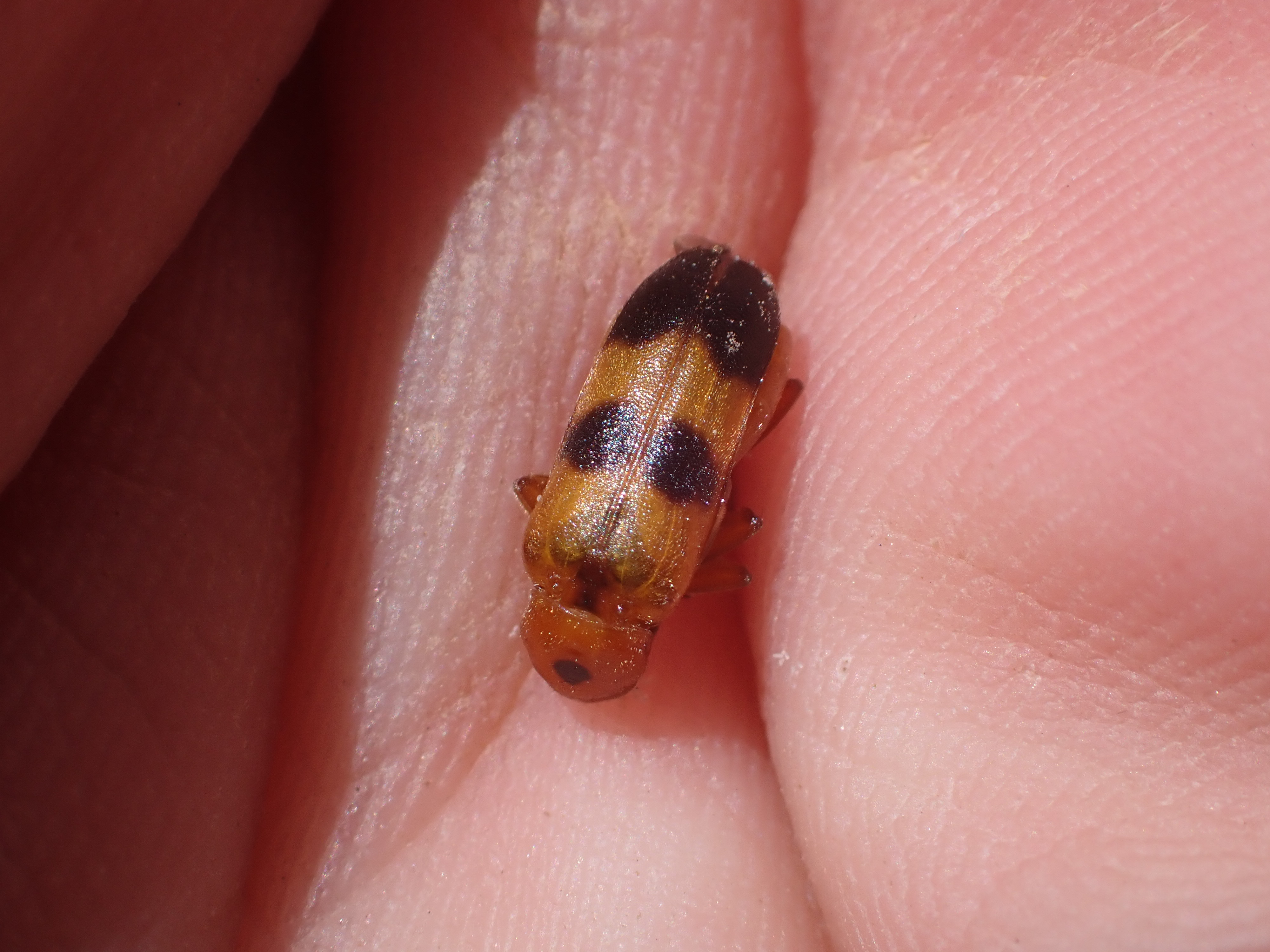

Supplement: Supplementary material 18 — Nemognatha chrysomelina (VLA_1518) [file bdj-13-e174504-s018.jpg]

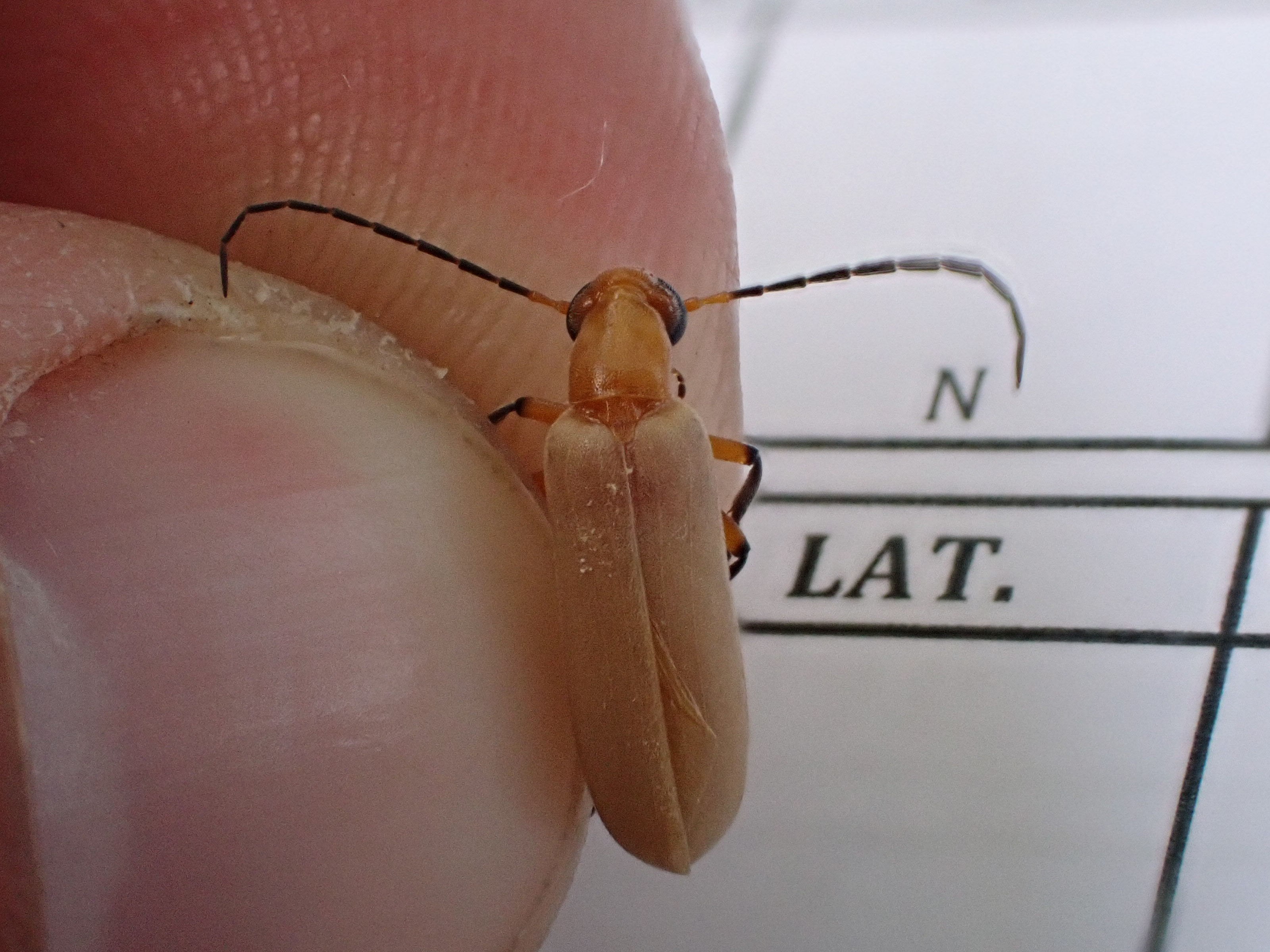

Supplement: Supplementary material 19 — Zonitoschema iranica (VLA_2092) [file bdj-13-e174504-s019.jpg]

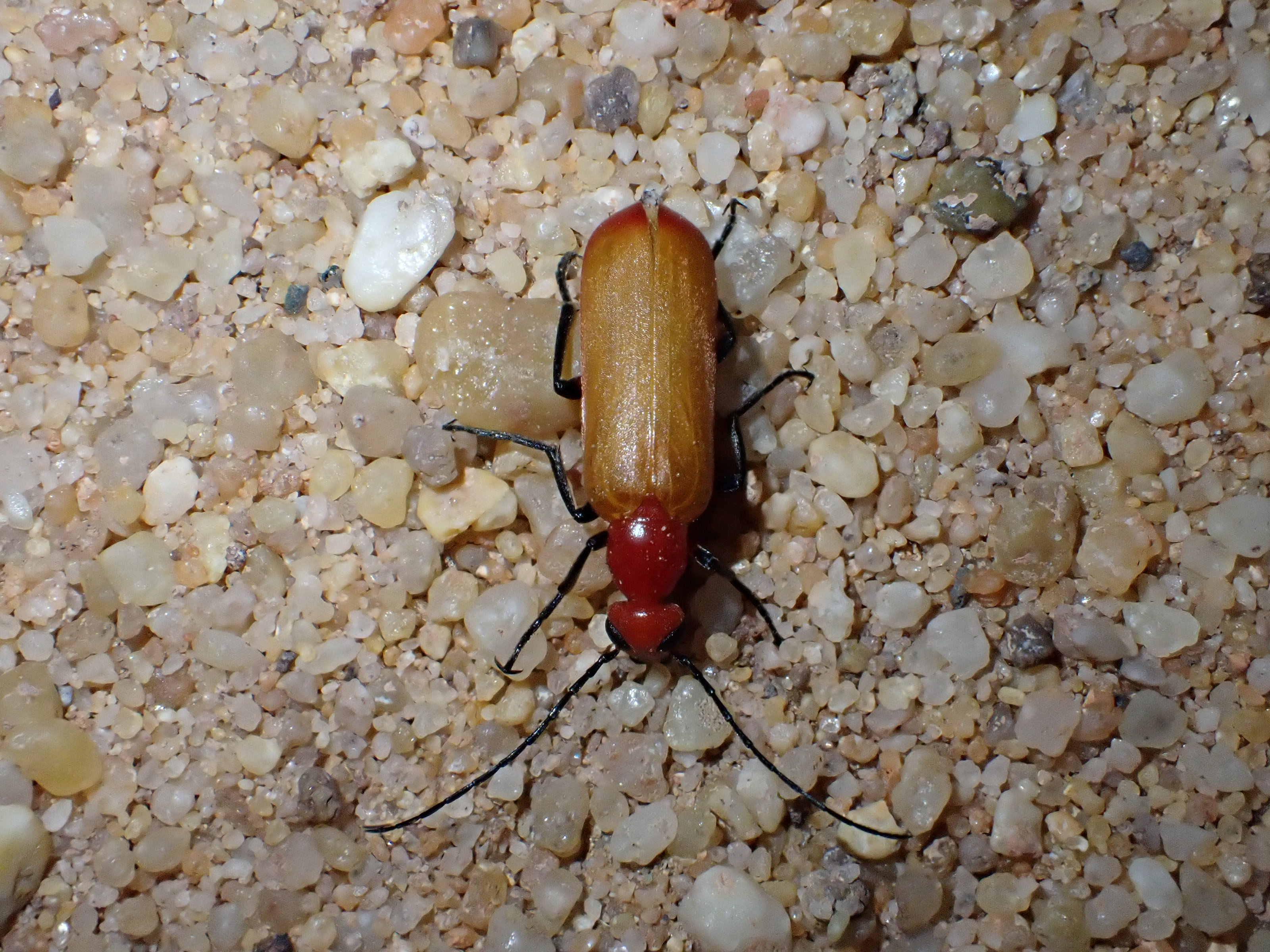

Supplement: Supplementary material 20 — Zonitoschema rubricolor (VLA_2282) [file bdj-13-e174504-s020.jpg]

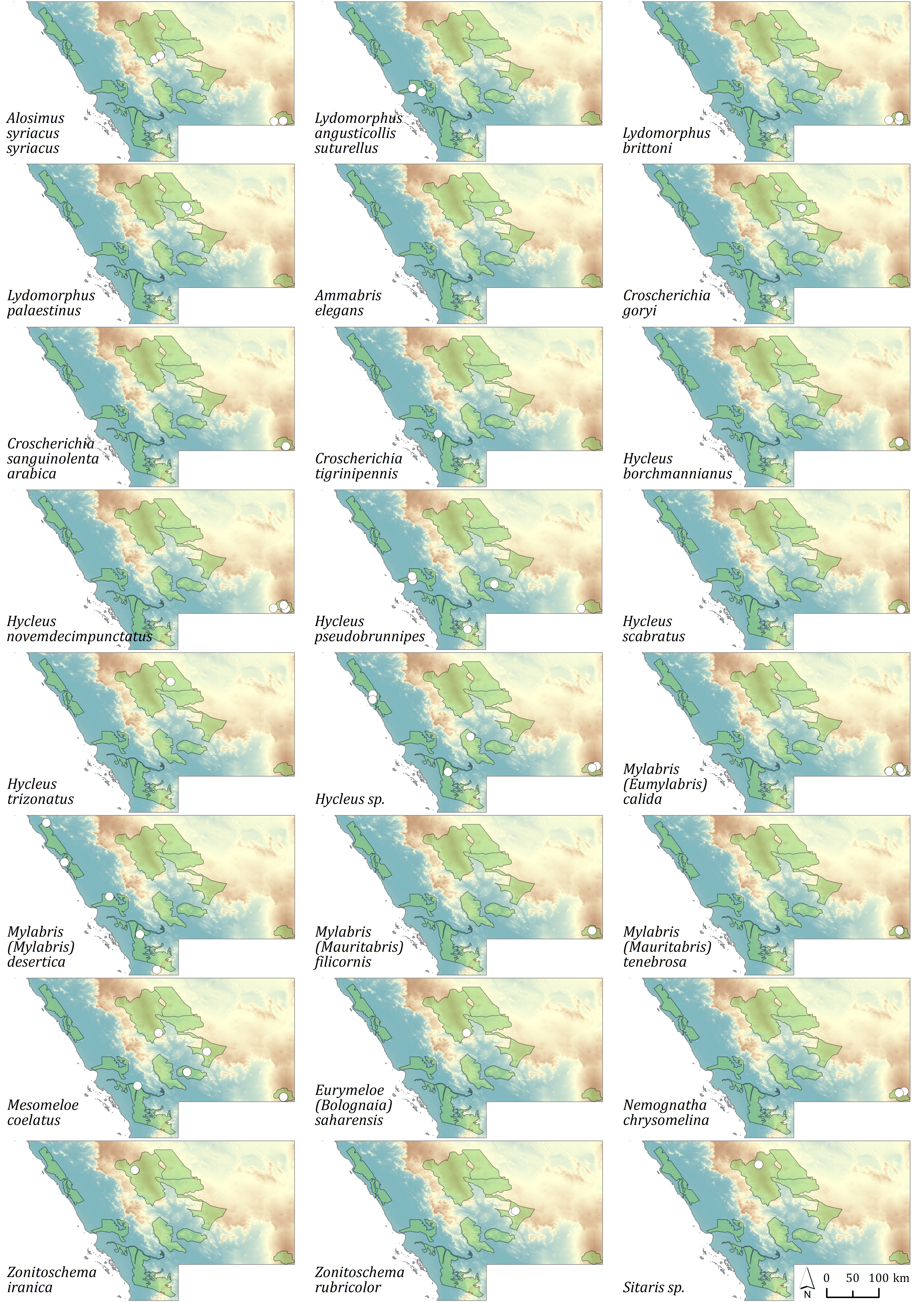

Supplement: Supplementary material 21 — Meloidae species distribution in the study area [file bdj-13-e174504-s021.jpg]

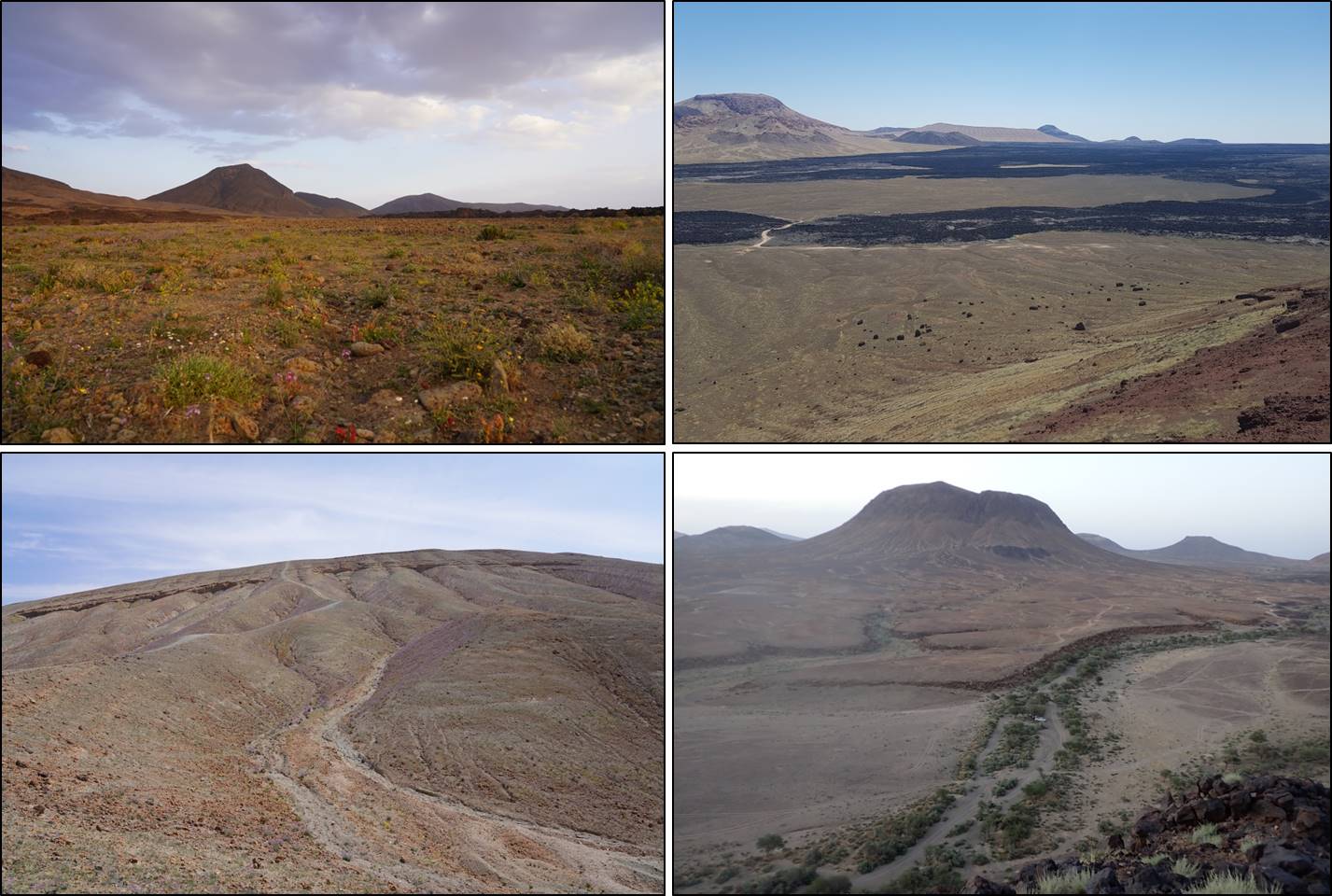

Supplement: Supplementary material 22 — Sites in Khaybar White Volcano Geopark. [file bdj-13-e174504-s022.jpg]
